# A novel, high-performance, low-volume, rapid luciferase immunoprecipitation system (LIPS) assay to detect autoantibodies to zinc transporter 8

**DOI:** 10.1093/cei/uxad139

**Published:** 2023-12-27

**Authors:** Claire L Williams, Ilaria Marzinotto, Cristina Brigatti, Kathleen M Gillespie, The BOX Study Group, Isabel Wilson, Rachel Aitken, Clare Megson, Chitrabhanu Ballav, Atanu Dutta, Michelle Russell-Taylor, Rachel Besser, James Bursell, Shanthi Chandran, Milton Keynes, Sejal Patel, Anne Smith, Manohara Kenchaiah, Gomathi Margabanthu, Foteini Kavvoura, Chandan Yaliwal, Vito Lampasona, Alistair J K Williams, Anna E Long

**Affiliations:** Translational Health Sciences, Bristol Medical School, University of Bristol, Southmead Hospital, Bristol, UK; San Raffaele Diabetes Research Institute, IRCCS San Raffaele Scientific Institute, Milan, Italy; San Raffaele Diabetes Research Institute, IRCCS San Raffaele Scientific Institute, Milan, Italy; Translational Health Sciences, Bristol Medical School, University of Bristol, Southmead Hospital, Bristol, UK; Bucks Healthcare Trust, UK; Oxford University Hospitals Trust UK, UK; University Hospital, UK; University Hospital, UK; University Hospital, UK; Wexham Park Hospital, UK; Northampton General Hospital, UK; Northampton General Hospital, UK; Kettering General Hospital, UK; Royal Berkshire Hospital, UK; Royal Berkshire Hospital, UK; San Raffaele Diabetes Research Institute, IRCCS San Raffaele Scientific Institute, Milan, Italy; Translational Health Sciences, Bristol Medical School, University of Bristol, Southmead Hospital, Bristol, UK; Translational Health Sciences, Bristol Medical School, University of Bristol, Southmead Hospital, Bristol, UK

**Keywords:** autoantibodies, immunoassay, LIPS, method development, risk prediction, type 1 diabetes

## Abstract

**Background:**

Zinc transporter 8 autoantibodies (ZnT8A) are thought to appear close to type 1 diabetes (T1D) onset and can identify high-risk multiple (≥2) autoantibody positive individuals. Radiobinding assays (RBA) are widely used for ZnT8A measurement but have limited sustainability. We sought to develop a novel, high-performance, non-radioactive luciferase immunoprecipitation system (LIPS) assay to replace RBA.

**Methods:**

A custom dual C-terminal ZnT8 (aa268-369; R325/W325) heterodimeric antigen, tagged with a Nanoluciferase^TM^ (Nluc-ZnT8) reporter, and LIPS assay was developed. Assay performance was evaluated by testing sera from new onset T1D (*n* = 573), healthy schoolchildren (*n* = 521), and selected first-degree relatives (FDRs) from the Bart’s Oxford family study (*n* = 617; 164 progressed to diabetes).

**Results:**

In new-onset T1D, ZnT8A levels by LIPS strongly correlated with RBA (Spearman’s *r* = 0.89; *P* < 0.0001), and positivity was highly concordant (94.3%). At a high specificity (95%), LIPS and RBA had comparable assay performance [LIPS pROC-AUC(95) 0.032 (95% CI: 0.029–0.036); RBA pROC-AUC(95) 0.031 (95% CI: 0.028–0.034); *P* = 0.376]. Overall, FDRs found positive by LIPS or RBA had a comparable 20-year diabetes risk (52.6% and 59.7%, respectively), but LIPS positivity further stratified T1D risk in FDRs positive for at least one other islet autoantibody detected by RBA (*P* = 0.0346).

**Conclusion:**

This novel, high-performance, cheaper, quicker, higher throughput, low blood volume Nluc-ZnT8 LIPS assay is a safe, non-radioactive alternative to RBA with enhanced sensitivity and ability to discriminate T1D progressors. This method offers an advanced approach to current strategies to screen the general population for T1D risk for immunotherapy trials and to reduce rates of diabetic ketoacidosis at diagnosis.

## Teaser

The LIPS method for measurement of autoantibodies to zinc transporter 8 (ZnT8A) can replace the radiobinding assay. This method also offers an advanced approach to current strategies to screen the general population for type 1 diabetes risk for immunotherapy trials and to reduce rates of diabetic ketoacidosis at diagnosis.

## Introduction

Type 1 diabetes (T1D) results from progressive autoimmune-mediated destruction of insulin-producing beta-cells in pancreatic islets. The most effective biomarkers used to predict the development of T1D are four major islet autoantibodies that recognize beta-cell antigens with high specificity: autoantibodies to endogenous insulin (IAA), glutamic acid decarboxylase 65 (GADA), islet antigen 2 (IA-2A), and zinc transporter 8 (ZnT8A). Using these four markers, >90% of islet autoimmunity can be detected at the clinical onset of T1D [1, 2], but these islet autoantibodies can be detected many years before diagnosis [3]. During the prodrome of T1D, the development of islet autoantibodies appears to occur sequentially, spreading from one beta-cell antigen to multiple antigens [4–6]. The risk of T1D correlates with the number of islet autoantibodies [7] and is highest in individuals with multiple autoantibodies (≥2), which confers up to 80% risk within 10–15 years in childhood [3]. Despite the well-described islet autoantibody staging of T1D risk [8], progression rates can vary from months to decades [9].

Identified in 2007, ZnT8A are among the most recently discovered islet autoantibody markers and are present in ~60–80% of new-onset T1D, dependent on age at onset [10]. Data from some birth-cohort studies of European descent have shown that in preclinical T1D, ZnT8A alongside IA-2A, are often not present at primary seroconversion and appear closer to onset, in children already positive for either GADA and/or IAA [11, 12]. Therefore, ZnT8A can be used to identify individuals at the greatest risk of progressing to T1D independent of other islet autoantibodies [11, 13–15]. Thus, accurate detection of ZnT8A is pivotal in identifying high-risk individuals for enrolment in secondary intervention clinical trials aimed at delaying progression to T1D.

Current methods to detect ZnT8A use C-terminal ZnT8 (aa268-369) which includes a common single nucleotide polymorphism (SNP) site at aa325 that encodes arginine (R325) or tryptophan (W325). This polymorphic variant forms two major epitopes of ZnT8A giving rise to three specificities: R325-specific (ZnT8RA), W325-specific (ZnT8WA), and non-specific ZnT8A that bind ZnT8 independent of this SNP site [16]. Antigen-specific radiobinding assays (RBAs) are well described and widely utilized to measure islet autoantibodies with individual laboratories electing to immunoprecipitate ZnT8A in serum utilizing monomeric (ZnT8-R325 or ZnT8-W325) or heterodimeric (ZnT8-R325 + ZnT8-W325) [^35^S]-methionine labelled ZnT8 peptides. Whilst RBAs are still often regarded as the “gold-standard” method for islet autoantibody measurement, the use of radioisotopes is costly, labour-intensive, tightly regulated, has limited long-term sustainability, and cannot be applied to large-scale population studies or be easily implemented into clinical practice. As > 80% of new cases occur outside of T1D-affected families [17], an active part of current research is to develop high-performance, low-volume, high throughput, cost-effective, and non-radioactive assays with the capacity for automation to enable screening for T1D in the general population.

We previously developed a NanoLuciferase^TM^ (Nluc)-IAA LIPS method which not only showed high concordance with RBA but had enhanced sensitivity in new-onset T1D and predictive utility in first-degree relatives (FDRs) that subsequently progressed to T1D [18]. Therefore, we optimized a novel Nluc-tagged ZnT8-R325 + ZnT8-W325 dual heterodimer (Nluc-ZnT8) LIPS fluid-phase immunoassay using the superior Nluc/furimazine reaction [19] and, evaluated its performance and predictive utility for T1D in a large population-based UK T1D family cohort.

## Materials and methods

### Study subjects

#### New-onset type 1 diabetes and first-degree relatives from the Bart’s-Oxford family study

The well-characterized population-based Bart’s Oxford (BOX) family study, established in 1985, has recruited and prospectively followed individuals under 21 years of age with newly diagnosed T1D and their FDRs within the former Oxford Regional Health Authority in the UK [20]. The BOX study is currently approved by the South Central—Oxford C. Research Ethics Committee. Participants provided informed, written consent and the study was performed according to the principles of the Declaration of Helsinki.

Serum samples from BOX participants were selected based on available historical data from ZnT8-R325 and ZnT8-W325 monomeric RBAs and sufficient sample volume for testing Table 1. A total of 573 new-onset T1D samples (<3 months of diagnosis) were selected where sera were available to evaluate whether Nluc-ZnT8 LIPS had improved sensitivity [RBA positive *n* = 388 (67.7%); RBA negative *n* = 185 (32.3%); 320 male (55.9%); median age at onset 11.3 years (range 1.0–54.9)]. FDRs of people with T1D are followed annually for the development of diabetes by questionnaire and were predominantly tested for ZnT8A in individuals positive for at least one other autoantibody [GADA/IA-2A/IAA by RBA and/or islet cell antibody (ICA; >20 Juvenile Diabetes Foundation Units (JDF-U) assay, previously described [21–24]]. The first available sample based on RBA ZnT8A status from FDRs was selected [*n* = 617; 315 (51.1%); median age at sample 32.4 years (range 0.4–66.0); median follow-up 17.1 years (range 0.2–36.8)], which included 164 (26.3%) FDRs that progressed to diabetes (any classification) over study follow-up [*n* = 99 males (60.4%); median follow-up 9.5 years (range 0.2–32.5); median age at sampling 38.5 years (range 1.4–59.4); median age at diagnosis 49.3 years (range 3.3–75.2). From questionnaire responses, of 31 RBA ZnT8A positive FDRs, 22 (71.0%) were diagnosed with T1D, 8 (25.8%) with type 2 diabetes (T2D), and 1 (3.15%) with other forms of diabetes. Similarly, of 133 RBA ZnT8A negative FDRs, 23 (17.3%) were diagnosed with T1D, 108 (81.2%) with T2D, and 2 (1.5%) with other forms of diabetes. FDRs who progressed to diabetes were oversampled independent of RBA ZnT8A status (positive/negative) to examine whether Nluc-ZnT8 LIPS had improved predictive utility for discriminating T1D risk.

**Table 1.** New-onset T1D and FDRs selected from the BOX family study

	New-onset T1D *n* = 573		RBA ZnT8A positive FDRs *n* = 53		RBA ZnT8A negative FDRs *n* = 564	
	RBA ZnT8A positive *n* = 388	RBA ZnT8A negative *n* = 185	Progressors *n* = 31	Non-progressors *n* = 22	Progressors *n* = 133	Non-progressors *n* = 431
Male/female (% male)	222/166 (57.2)	98/87 (53.0)	13/18 (41.9)	10/12 (45.5)	86/47 (64.7)	206/225 (47.8)
Median age at onset (years; range)	11.2 (1.3–20.8)	11.7 (1.0–54.9)	30.6 (3.3–73.1)	—	50.4 (5.1–75.2)	—
Median age at sample (years; range)	11.2 (1.3–20.8)	11.7 (1.1–54.7)	20.0 (2.1–44.4)	18.8 (2.6–60.0)	39.2 (1.4–59.4)	26.5 (0.4–66.0)
Median diabetes duration or follow-up (years; range)	0.0 (−0.2 to 0.1)	0.0 (−0.2 to 0.2)	6.0 (0.3–32.5)	22.3 (3.2–33.6)	10.3 (0.2–28.9)	20.3 (0.4–36.8)
ZnT8RA (%)	347 (89.4)	—	28	20	—	—
ZnT8WA (%)	291 (75.0)	—	24	16	—	—
ZnT8R/WA (%)	388 (100.0)	—	31 (100.0)	22 (100.0)	—	—
ZnT8RA-specific (%) ZnT8WA-specific (%) ZnT8A non-specific (%)	97 (25.0) 41 (10.6) 250 (64.4)	—	7 (22.6) 3 (9.7) 21 (67.7)	6 (27.3) 2 (9.1) 14 (63.6)	—	— — —
Single autoantibody positive ± ICA^*^ (%)	8 (2.1)	53 (28.6)	1 (3.2)	1 (4.5)	29 (21.8)	197 (45.7)
Positive for multiple (≥2) autoantibodies	380 (97.9)	114 (61.6)	30 (96.8)	21 (95.5)	14 (10.5)	14 (3.2)
GADA (%)	325 (83.8)	144 (77.8)	28 (90.3)	19 (86.4)	29 (21.8)	119 (27.6)
IA-2A (%)	324 (83.5)	106 (57.3)	18 (58.1)	12 (54.5)	8 (6.0)	20 (4.6)
IAA/n^**^ (%)	176/234(75.2)	71/121 (58.7)	16 (51.6)	10/22 (83.3)	22 (16.5)	88 (20.4)
ICA^*^/*n* with data (%)	156/215 (72.6)	51/123 (41.5)	7/27 (25.9)	7/20 (35.0)	5/104 (4.8)	31/401 (7.7)
Biochemical autoantibody negative ± ICA^*^ (%)	—	18 (9.7)	0 (0.0)	—	90 (67.7)	220 (51.0)

^*^Islet cell antibody (ICA) considered positive > 20 juvenile diabetes foundation (JDF) used as positivity threshold in the European Nicotinamide Intervention Trial (ENDIT) [21]; ICA positivity with 1 other biochemical autoantibody (GADA, IA-2A, IAA, or ZnT8A) has a comparable risk of T1D to single biochemical autoantibody positives.

^**^IAA data considered if the sample was taken before onset or within 2 weeks of diagnosis before exogenous insulin treatment.

#### Anonymized healthy schoolchildren for positivity thresholds

Positivity thresholds in a population of 523 anonymized healthy schoolchildren [*n* = 279 (53.4%) male; median age 11.3 years (range 9.0–13.8); 464 (88.7%) Caucasian ethnicity], described previously [25], were established for ZnT8-R325 and ZnT8-W325 monomeric RBAs. Samples with sufficient volume were used to assess assay performance and establish the Nluc-ZnT8 LIPS positivity threshold following assay optimization [*n* = 521/523 (99.6%)].

### Detection of ZnT8A by monomeric (ZnT8-R325/ZnT8-W325) RBA

Recombinant ZnT8 antigens encoded in a modified pCMVTnT^TM^ vector (Promega, WI, USA) were kindly supplied by Dr V. Lampasona (Milan, Italy). Autoantibodies to ZnT8 [aa268-369; ZnT8-R325 and ZnT8-W325 peptides] were detected by monomeric RBAs described previously [26]. An in-house logarithmic standard curve made from eight serial dilutions of a high T1D pool of ZnT8RA/ZnT8WA positive sera was used to determine ZnT8A binding expressed as arbitrary units (AU). The positivity threshold at the 97.5th percentile of 523 healthy schoolchildren was 1.8AU. In the 2020 Islet Autoantibody Standardisation Program (IASP2020) workshop, the adjusted sensitivity at 95% specificity (AS95) of ZnT8-R325 RBA and ZnT8-W325 RBA was 70.0% and 56.0%, respectively.

### Detection of ZnT8A by the Nluc-ZnT8 LIPS assay

#### Expression and preparation of recombinant Nluc-ZnT8-R325 + W325 dual heterodimer antigen

One microgram of Nluc-ZnT8 dual heterodimer antigen (ZnT8-R325 + ZnT8-W325-Nluc-ZnT8-R325 + ZnT8-W325; Supplementary Fig. S1), sub-cloned into a modified pCMVTnT^TM^ vector (Promega; kindly supplied by V. Lampasona), was incubated for 2 h at 30°C with reagents from the TnT® SP6 *in vitro*-coupled transcription/translation system (Promega). At room temperature (RT), the neat reaction mixture was initially diluted with 250 µl phosphate-buffered saline with 0.1% Tween-20 (PBST) and gently mixed by inverting. Serial 10-fold dilutions using 2 µl from the initial dilution were prepared in PBST (1:250, 1:2500, and 1:25 000) and mixed by inverting. In duplicate, 25 µl of all serial dilutions was added to a 96-well Optiplate^TM^ (Perkin Elmer, MA, USA) and after the addition of 40 µl of Nano-Glo® Furimazine substrate (Promega; 1:50 as per manufacturer’s instructions), the bioluminescence emission expressed in light unit equivalents (LU) was detected (2 s/well) by a Bertold Centro XS3 luminometer (Bertold Technologies GmBH and Co. KG, Bad Wildbad, Germany). After multiplication by the dilution factors, a typical reaction yielded 3 × 10^8^ to 7 × 10^8^ LU/µl. The remaining initial dilution was aliquoted into 10 µl single-use aliquots and stored at −70°C. Before use, single-use aliquots were thawed at RT, diluted 1:100 in Tris-buffered saline with 0.5% Tween 20 (TBST-0.5%), and filtered through a PVDF 0.45µM Millex-SV syringe filter (Merck, Darmstadt, Germany).

#### The Nluc-ZnT8 LIPS assay methodology

Filtered Nuc-ZnT8 dual heterodimer antigen was further diluted to 4.0 × 10^6^ (±0.2 × 10^6^) LU/25 µl in TBST-0.15% Tween-20 (TBST-0.15%). In duplicate, 1 µl of serum was plated into a 96-deep well plate (Sarstedt, Nümbrecht, Germany) and incubated with Nluc-ZnT8 dual heterodimer antigen for 2.5 h at RT shielded from light. The remainder of the LIPS methodology, previously described [27], was followed before the detection of bioluminescence. To a final volume of 30 µl, 40 µl/well of Nano-Glo® Furimazine substrate assay reagent (1:50 NanoGlo® Furimazine substrate further diluted to a final concentration of 1:150 with TBST-0.15%) was injected into each well immediately before LU determination by a Bertold Centro XS3 luminometer using a standardized detection protocol; inject 40 µl/well, shake 5 s/well, detect 2 s/well. The in-house logarithmic standard curve developed for the monomeric RBAs was used to determine and express ZnT8A binding as AUs. In the IASP2020 workshop, the AS95 was 78%.

### Data transformation and statistical analysis

Due to the presence of all ZnT8A specificities [16], the maximum AU derived from both monomeric RBA was used to compare Nluc-ZnT8 LIPS and RBA by Spearman’s rank correlation and Bland–Altman analysis. The sensitivity and specificity of the methods were assessed by the total area under the curve receiver operator curve (ROC–AUC) and partial ROC–AUC at 95% specificity (pROC–AUC(95)) analysis utilizing the new-onset T1D and healthy schoolchildren cohorts (pROC package for the R software [28]). Kaplan–Meir survival curve analysis was used to assess the predictive utility of the methods in FDRs and the Mantel–Cox log-rank test was used to compare survival between categories. Diabetes survival (%) between 5 and 20 years of study follow-up was reviewed. Unless stated otherwise, all graphs and statistical analysis were performed using the Prism v6 software (GraphPad Software, Inc., CA, USA; v. 9.1.0). An alpha value *P* < .05 was considered significant in all analyses.

## Results

A series of optimization experiments were conducted to improve assay throughput and reduce labour time and costs. These considered the Nluc-ZnT8 antigen construct (single vs. dual ZnT8-R325 + ZnT8-W325 heterodimer antigen; Supplementary Figs. S1 and S2), Nluc-ZnT8 antigen incubation length (Supplementary Fig. S2), Nluc-ZnT8 antigen preparation method (Supplementary Fig. S3), Nluc-ZnT8 antigen stability (Supplementary Fig. S4), and the Furimazine substrate concentration (Supplementary Fig. S5). Once optimized, the described method was used to investigate assay performance.

### Levels of ZnT8A detected by RBA and Nluc-ZnT8 LIPS were strongly correlated and ZnT8A positivity was highly concordant.

**Figure 1.**
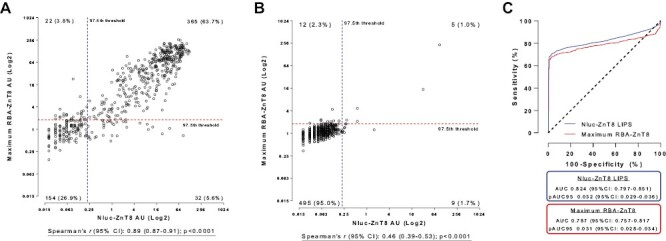
Nluc-ZnT8 LIPS and RBA were highly correlated and concordant, and at high specificities, assay sensitivity was comparable. Scatterplots comparing levels of ZnT8A binding (arbitrary units; AU) between Nluc-ZnT8 LIPS and monomeric RBAs in 573 newly diagnosed T1D patients (**A)** and 521 healthy schoolchildren (**B**) show that levels of ZnT8A detected by both assays were strongly correlated and ZnT8A positivity was highly concordant. The maximum AU obtained by monomeric RBA (ZnT8-R325/ZnT8-W325) was utilized for analysis as this improved the correlation between the two assays. (**C**) Receiver operator characteristic (ROC) analysis comparing new-diagnosed T1D and healthy schoolchildren cohorts indicated that the area under the curve (AUC) and assay sensitivity at 95% specificity was slightly improved in Nluc-ZnT8 LIPS [AUC 0.82 (95% CI 0.80–0.85, *P* < 0.0001); sensitivity 71.2%] compared to RBA [AUC 0.79 (95% CI 0.76–0.82); sensitivity 68.6%] (*P* = 0.0007 between methods). However, pAUC-ROC was comparable between the methods [Nluc-LIPS pROC-AUC 0.032 (95% CI: 0.029–0.036); RBA pROC-AUC 0.031 (95% CI: 0.028–0.034); *P* = 0.376]

In 573 new-onset T1D, levels of ZnT8A were highly correlated between RBA and Nluc-ZnT8 LIPS [*r*: 0.89 (95% CI: 0.87–0.90), *P* < 0.0001; Fig. 1A], but in both methods, ZnT8A levels did not correlate with age at onset (data not shown). Positivity for ZnT8A was also highly concordant between methods. Of 387 new-onset T1D found positive by RBA, Nluc-ZnT8 LIPS identified 94.3% (*n* = 365/387), but 5.7% (*n* = 22/387) were discrepant between methods. This could be due to lower-level and/or R325-specific/W325-specific responses; 95.5% (*n* = 21/22) were lower-level (<8AU), and 68.2% (*n* = 15/22) were R325-specific or W325-specific ZnT8A. Of these 22 discrepant samples, 1 (4.5%) was single autoantibody positive by ZnT8A RBA and 21 (95.5%) had at least one additional autoantibody. Of 186 new-onset T1D found negative by RBA, 82.8% (154/186) were concordant in Nluc-ZnT8 LIPS but Nluc-ZnT8 LIPS identified an additional 17.2% (32/186) new-onset T1D. Of these 32, 2 had no other autoantibody and 24 had at least one additional autoantibody, increasing single and multiple positivity by 6.3% and 18.8%, respectively. Of all discrepant samples between methods (*n* = 54), 83.3% (*n* = 45) were positive for at least one other islet autoantibody (GADA/IA-2A/IAA) and therefore, would be identified in conjunction with other tests. More detail on discrepant samples is described in Supplementary Table S1. Overall, Bland–Altman analysis revealed that only 39 new-onset T1D (6.8%) were outside the 95% CI of agreement with Nluc-ZnT8 LIPS reporting lower ZnT8A levels compared with RBA (Supplementary Fig. S6A and S6B).

In 521 healthy schoolchildren, levels of ZnT8A were also correlated between RBA and Nluc-ZnT8 LIPS [*r*: 0.46 (95% CI: 0.39–0.53), *P* < 0.0001; Fig. 1B] with ZnT8A status concordant in 96.0% (*n* = 500/521) between methods. Of 21 discrepant samples, 2.3% (12/521) were found positive by RBA but negative by Nluc-ZnT8 LIPS, and 1.7% (9/521) were found negative by RBA but were positive by Nluc-ZnT8 LIPS. None of the 21 discrepant samples were found positive for other islet autoantibodies and Bland–Altman analysis revealed that only 2 of all 521 (0.4%) healthy schoolchildren were outside the 95% CI of agreement (Supplementary Fig. 6C/D). The threshold for positivity for Nluc-ZnT8 LIPS was placed at the 97.5th percentile of this cohort (0.22AU).

### The Nluc-ZnT8 LIPS assay and RBA have comparable sensitivity at high specificity.

Using the established 97.5th percentile threshold and the two cohorts of samples from new-onset T1D and healthy schoolchildren, the AUC-ROC for Nluc-ZnT8 LIPS was 0.824 (95% CI: 0.797–0.851, *P* < 0.0001) and the AUC-ROC for RBA was 0.79 (95% CI: 0.757–0.817, *P* < 0.0001) (Fig. 1C). There was strong evidence to suggest a difference in AUC-ROC between the methods (*P* = 0.0007), offering an assay sensitivity at 95% specificity of 71.2% versus 68.6% in RBA. However, pROC-AUC(95) between methods were comparable [Nluc-ZnT8 LIPS pROC-AUC(95) 0.032 (95% CI: 0.029–0.036); RBA pROC-AUC(95) 0.031 (95% CI: 0.028–0.034), *P* = 0.376].

### The Nluc-ZnT8 LIPS assay discriminates diabetes risk in first-degree relatives positive for other islet autoantibodies detected by RBA

**Figure 2.**
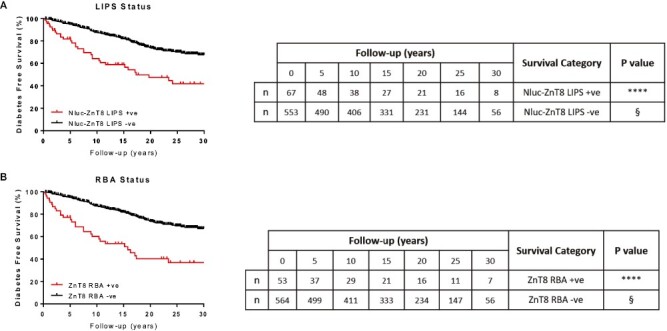
Measurement of ZnT8A by Nluc-ZnT8 LIPS or RBA has comparable diabetes risk in FDRs. § Reference category for Mantel–Cox log-rank tests. ****P < 0.0001. Positivity for ZnT8A measured by Nluc-ZnT8 LIPS (**A**) or RBA (**B**) had a much higher diabetes risk than those found ZnT8A negative by either method (*P* < 0.0001). Overall diabetes risk is comparable in FDRs found positive or negative by either Nluc-ZnT8 LIPS or RBA however, Nluc-ZnT8 LIPS identified a greater number of at-risk individuals at first sampling or first ZnT8A positive sample (67 vs. 53)

Overall, relatives found positive by either Nluc-ZnT8 LIPS or monomeric RBAs had a comparable 20-year diabetes risk between 52.6% (95% CI: 38.7–65.3) and 59.7% (95% CI: 45.2–73.8), respectively (*P* > 0.05, Fig. 2A and B; Table 2). Similarly, relatives found negative by either method had a comparable 20-year diabetes risk ~26.0% [LIPS 26.0% (95% CI: 21.6–30.0); RBA 26.1 (95% CI: 21.7–30.0)].

**Table 2.** Diabetes risk in FDRs over 20-years of follow-up in the BOX study

Group	Stratification	*n* FDRs	5-year diabetes risk (%; 95% CI)	10-year diabetes risk (%; 95% CI)	15-year diabetes risk (%; 95% CI)	20-year diabetes risk (%; 95% CI)
**All relatives**	LIPS +ve	67	18.3 (6.6–25.7)	35.8 (22.3–46.5)	41.2 (27.6–52.4)	52.6 (38.7-65.3)
	LIPS −ve	553	5.1 (2.8–6.6)	12.5 (9.2–15.1)	18.0 (14.2–21.1)	26.0 (21.6-30.0)
**All relatives**	RBA +ve	53	22.7 (8.9–31.8)	39.9 (24.9–52.0)	46.3 (31.4–59.1)	59.7 (45.2-73.8)
	RBA –ve	564	5.0 (2.8–6.5)	12.7 (9.5–15.3)	18.1 (14.4–21.3)	26.1 (21.7-30.0)
**RBA + ve**	LIPS +ve	49	24.5 (9.9–34.3)	45.8 (30.2–59.0)	53.6 (38.2–67.6)	59.8 (44.7–74.4)
	LIPS –ve	4	0 (–)	0 (–)	0 (–)	50.0 (58–84.5)
**RBA –ve**	LIPS +ve	18	0 (–)	13.8 (−17.3 to 24.0)	13.8 (−17.3 to 24.0)	32.2 (–1.3-51.2)
	LIPS –ve	546	5.3 (3.0–6.9)	12.6 (9.4–15.3)	18.2 (14.4–21.5)	25.9 (21.4–29.8)
**Single RBA Aab +ve**	LIPS +ve	22	9.3 (−13.8 to 16.2)	15.0 (−9.9 to 24.9)	20.7 (−5.1 to 33.1)	28.6 (0.2–44.6)
	LIPS –ve	216	3.8 (0.1–5.8)	8.2 (3.3–11.3)	9.6 (4.3–13.0)	14.6 (7.9–19.3)
**Multiple RBA Aab +ve**	LIPS +ve	43	23.5 (7.7–33.6)	45.4 (28.5–59.4)	50.8 (34.3–65.4)	64.9 (49.4–80.8)
	LIPS −ve	24	8.3 (−12.7 to 14.5)	32.2 (8.2–47.6)	37.4 (13.4–54.2)	56.2 (33.4–76.9)

Diabetes risk (%; 95% confidence interval (CI)) at 5-year increments of study follow-up. A maximum of 20 years follow-up was reviewed where ≥100 FDRs remained. Across all Kaplan–Meier survival analysis, there was no clear difference in the performance of LIPS and RBA across all 5-year increments of study follow-up, except in relatives found single autoantibody positive by LIPS or by GADA/IA-2A/IAA RBAs.

**Figure 3.**
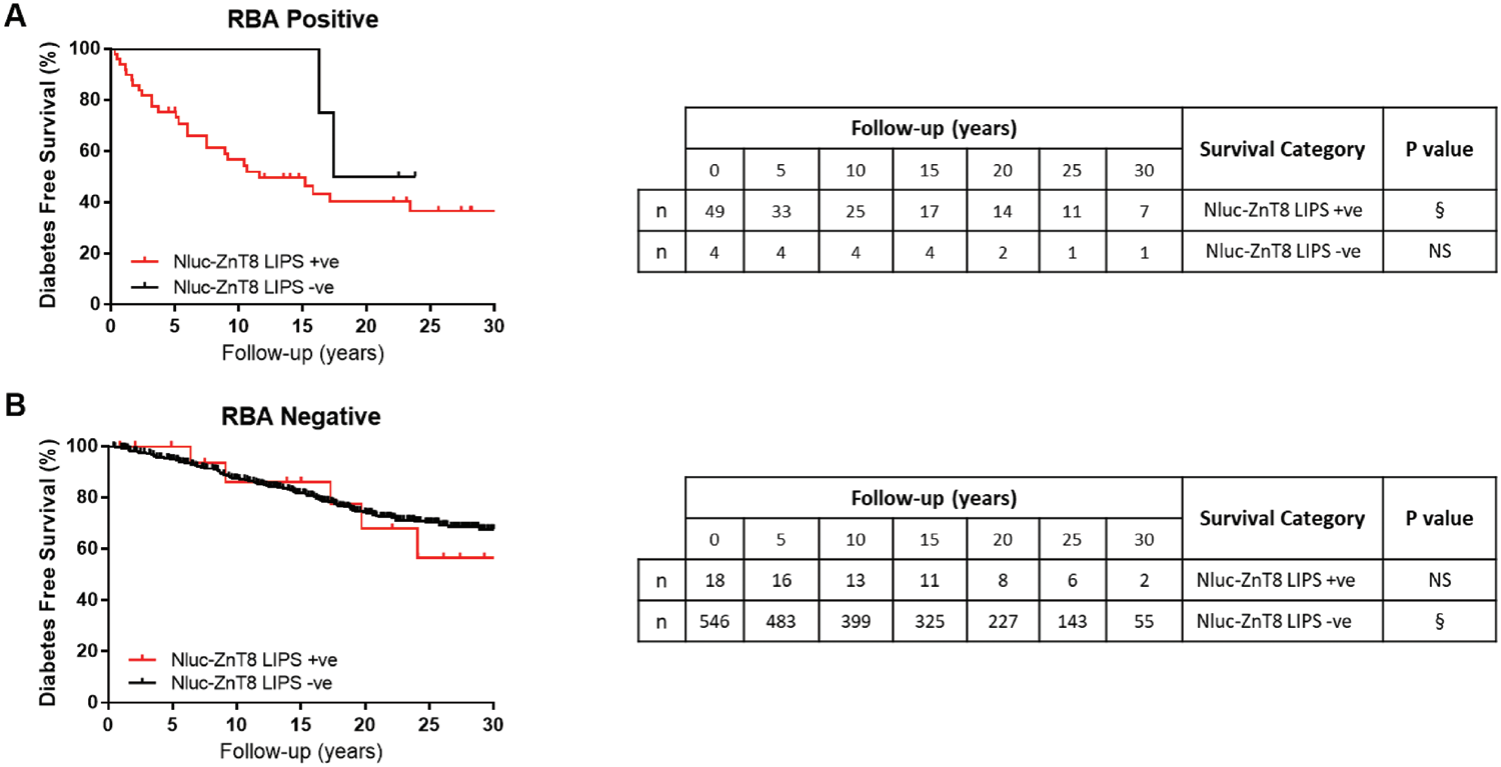
Nluc-ZnT8 LIPS ZnT8A status does not identify FDRs with higher diabetes risk when stratified by RBA ZnT8A status. § Reference category for Mantel–Cox log-rank tests. NS: not significant. (**A**) Nluc-ZnT8 LIPS status in FDRs found positive by RBA. FDRs identified as positive in both Nluc-ZnT8 LIPS and RBA had the highest 20-year risk at 59.8%. Only four FDRs found positive by RBA were found negative by Nluc-ZnT8 LIPS but only two of these (50%) progressed to diabetes within 20 years of follow-up. Due to limited numbers, these two curves could not be robustly compared. (**B**) Nluc-ZnT8 LIPS status in FDRs found negative by RBA. Nluc-ZnT8 LIPS identified a small subset of additional FDRs that progressed to diabetes than RBA (20-year diabetes risk of 32.2%), but this was of comparable risk to relatives found negative by both methods (20-year diabetes risk of 25.9.%)

In relatives that were positive for both Nluc-ZnT8 LIPS and monomeric RBA, the 20-year diabetes risk was 59.8% (95% CI 44.7–74.4) (Fig. 3A; Table 2). There were only four relatives that the monomeric RBAs found positive that the LIPS assay did not identify, and, of these, two (50%) relatives slowly progressed to diabetes over a 20-year follow-up. Due to limited numbers, these two curves could not be robustly compared. In relatives that tested negative by both monomeric RBAs, Nluc-ZnT8 LIPS identified a small subset of 18 relatives that had a 20-year diabetes risk of 32.2% (95% CI −1.3 to 51.2) [Fig. 3B; Table 2; 5/18 relatives later progressed to diabetes (range 6.1–24.1 years after sampling)], but this was not different from relatives found negative by both methods [20-year diabetes risk of 25.9.% (95% CI: 21.4–29.8), *P* > 0.05].

**Figure 4.**
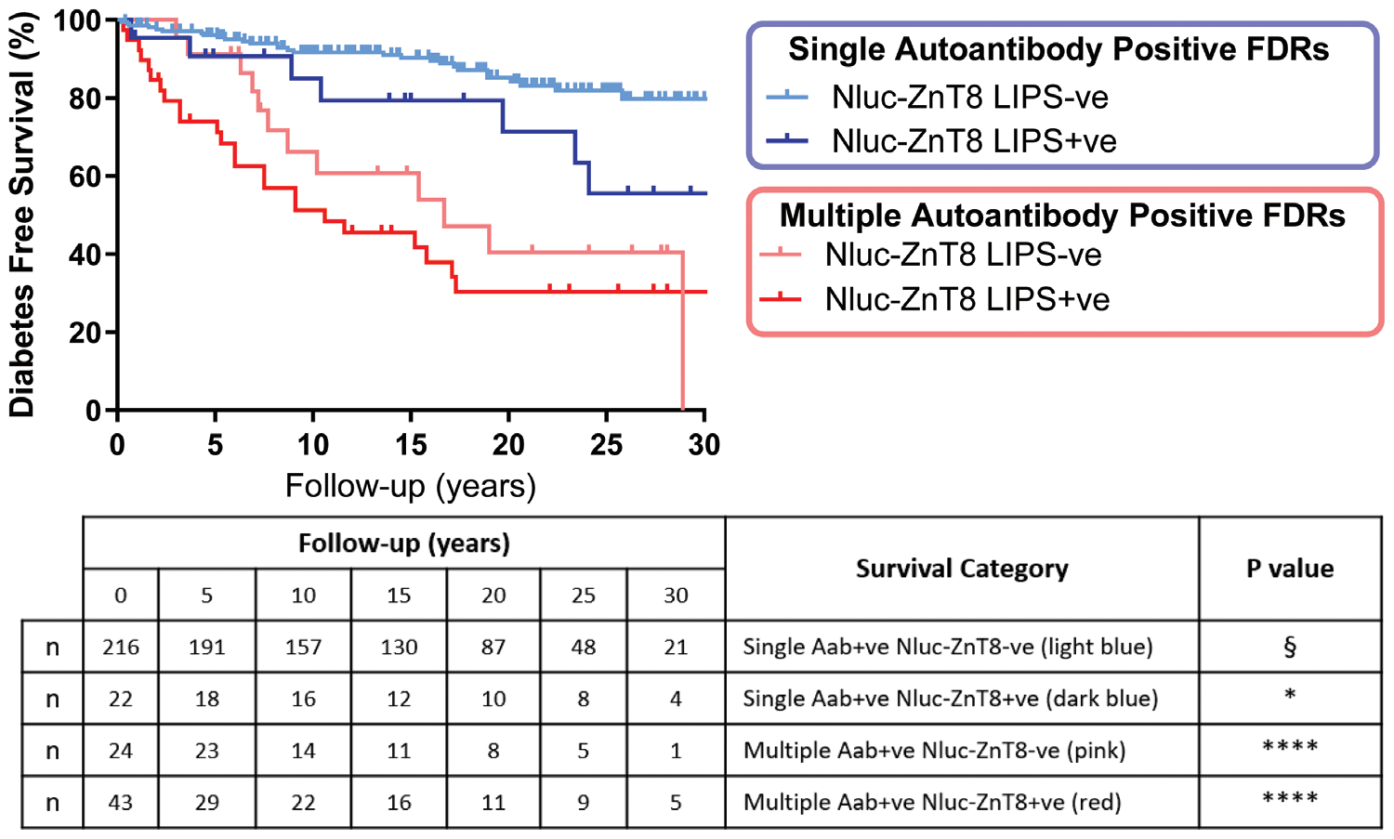
Positivity of ZnT8A by Nluc-ZnT8 LIPS further stratifies diabetes risk in FDRs positive for other islet autoantibodies. § Reference category for Mantel–Cox log-rank tests. *P < 0.05; ****P < 0.0001. FDRs classified as single or multiple autoantibody positive considering GADA/IA-2A/IAA islet autoantibodies determined by RBA stratified according to Nluc-ZnT8 LIPS status. Nluc-ZnT8 LIPS positivity compared to negativity confers a higher 20-year diabetes risk in single autoantibody positive FDRs (28.6% versus 14.6%, *P* = 0.0346). Similarly, Nluc-ZnT8 LIPS positivity in multiple autoantibody FDRs conferred the highest 20-year diabetes risk (64.9%) however, this was not significantly different to FDRs found multiple autoantibody positive considering other islet autoantibodies (56.2%) (*P* > 0.05). Of the 7 single and 26 multiple autoantibody positive individuals that were additionally identified by Nluc-ZnT8 LIPS, a total of 2/7 (28.6%) and 11/26 (42.3%), respectively, rapidly progressed to T1D within 5 years of sampling

Considering the detection of GADA, IA-2A, or IAA by RBA, relatives found single autoantibody positive by Nluc-ZnT8 LIPS had a greater 20-year diabetes risk [28.6% (95% CI: 0.2–44.6)] compared with those found single autoantibody positive for other islet autoantibodies [14.6% (95% CI: 7.9–19.3), *P* = 0.0346] (Fig. 4; Table 2). Relatives found multiple autoantibody positive by Nluc-ZnT8 LIPS or by other islet autoantibodies had a comparable 20-year risk [LIPS 64.9% (95% CI: 49.4–80.8); RBA 56.2% (95% CI: 33.4–76.9), *P* > 0.05] (Fig. 4; Table 2). Of the 7 single and 26 multiple autoantibody positive individuals that were additionally identified by Nluc-ZnT8 LIPS, a total of 2/7 (28.6%) and 11/26 (42.3%), respectively, rapidly progressed to T1D within 5 years of sampling. As expected, multiple autoantibody positive relatives, irrespective of islet autoantibody markers used, had a much higher diabetes risk than single autoantibody relatives (Fig. 4; *P* < 0.0001).

Across all Kaplan–Meir survival analysis, there was no clear difference in the performance of LIPS and RBA across all 5-year increments of study follow-up, except in relatives found single autoantibody positive by LIPS or GADA/IA-2A/IAA RBAs (Table 2).

## Discussion

A novel, low-volume, rapid, and high-performance fluid-phase Nluc-ZnT8 LIPS method to detect ZnT8RA/ZnT8WA simultaneously was developed and validated in a large cohort of healthy schoolchildren, new-onset T1D, and FDRs to replace the conventionally used fluid-phase RBA. Nluc-ZnT8 LIPS and RBA were highly correlated and concordant, and at high specificities, assay performance was similar. Overall diabetes risk was comparable between relatives who were found positive or negative by either method. When stratified by RBA status, Nluc-ZnT8 LIPS did identify a small additional subset of 18 relatives, but diabetes risk was similar to relatives found ZnT8A negative by both methods. Nluc-ZnT8 LIPS positivity was useful in further stratifying diabetes risk in relatives found positive for other islet autoantibodies measured by RBA across a wide age spectrum. Furthermore, Nluc-ZnT8 LIPS ranked among the top assays for ZnT8A measurement in IASP2020 and showed good inter-laboratory concordance (unpublished data). Accordingly, the data presented in this study provides strong evidence that the Nluc-ZnT8 LIPS assay is a suitable replacement for the RBA in the context of T1D.

The Nluc-ZnT8 LIPS methodology described in this study after several optimization steps resulted in a high-performance non-radioactive assay that has a one-day turnaround time, requires very low sample volume (2 µl/duplicate), and has a comparable methodology to the RBA with widely available reagents. This allows most laboratories who are running RBA or other similar immunoassay formats to easily implement the method with limited re-training requirements to dispense radiation use more readily. Nluc-ZnT8 LIPS assay optimization resulted in a quicker, cheaper, and more sustainable method than the RBA. The cost of reagents was reduced by decreasing the amount of protein A sepharose required, switching to an alternative method for antigen preparation, and further diluting the NanoGlo® Furimazine substrate. Currently, LIPS is approximately two-thirds cheaper than RBA and it is likely to become more cost-effective as the cost of radionuclides continues to increase. It is worth noting that ZnT8A levels were not comparable (but were highly correlated) between Nluc-ZnT8 LIPS and RBA. This probably indicates that the sera used to generate the standard curve behaved differently between methods compared with other samples tested, which resulted in a lower positivity threshold (0.22AU vs. 1.8AU in RBA at the 97.5th percentile). The Nluc-ZnT8 LIPS assay using a ZnT8-R325 + W325 dual heterodimer instead of monomeric ZnT8-R325/W325 peptides also allowed for simultaneous ZnT8A measurement without loss of sensitivity or specificity, which will also have cost- and time-saving implications to other laboratories with similar ZnT8A testing strategies. Collectively, these practical and cost-effective advantages did not negatively impact assay performance.

LIPS assay to detect GADA, IA-2A, ZnT8A, IAA, and autoantibodies to glycoprotein tetraspanin protein family member 7 (TSPAN7A) in T1D have been reported [18, 29–32]. These studies have utilized *Renilla* luciferase, *Gaussia* luciferase (Gluc), or Nluc and report either comparable or increased sensitivity compared with widely used assays [RBA/enzyme-linked immunosorbent assay (ELISA)]. Only one study has explored the measurement of ZnT8A using a ZnT8-R325 + W325 heterodimer by LIPS [31]. Comparable to the present study, Ustinova *et al*. (2014) describe a liquid-phase LIPS assay that can be conducted within 1 day and uses the same amount of neat sample, but there are a number of technical differences, particularly regarding the design and preparation of the antigen. First, the two designs of ZnT8 construct varies by both the luciferase and placement of the luciferase ZnT8-W325 + ZnT8-R325-Gluc heterodimer (Gluc-ZnT8) vs. ZnT8-R325 + ZnT8-W325-Nluc-ZnT8-R325 + ZnT8-W325 dual heterodimer (Nluc-ZnT8). Second, the Gluc-ZnT8 was overexpressed in an insect cell line (Tn5), which required a 55-h incubation and subsequent procedures to exclude insect-derived proteins before use. Whereas, this Nluc-ZnT8 method used a cell-free *in vitro* expression system which took ~2 h before use. While the Gluc-ZnT8 protocol produced comparably high antigen yields to the Nluc-ZnT8 protocol (>1 × 10^9^ LU/µl), the antigen was only stable for ~2 months at −80°C, but we observed stability ≥ 1-year at −80°C. Additionally, when the liquid-phase Gluc-ZnT8 LIPS assay was compared to the commercially available solid-phase RSR^TM^ Limited ELISA (Cardiff, UK) in new-onset T1D and controls, assay concordance was age-dependent (lower in children) and lower specificity (68–78%) was observed, even when a high antigen concentration (10–15 × 10^6^LU) was required to discriminate the populations. Age cut-offs in these populations were not reported; but using the RSR^TM^Limited ELISA, age-related positivity cut-offs for ZnT8A have been described in another study [33]. We did not observe age-dependent effects, but the majority of individuals with new-onset T1D studied were aged <21 years and healthy schoolchildren were of adolescent age. Nevertheless, our study includes one of the largest studies of ZnT8A prevalence in new-onset T1D (within 3 months of diagnosis) [1] and healthy control populations reported, in which we did not observe low sensitivity or specificity. This could be due to the many advantages of the NanoGlo® luciferase/substrate coupled system (Promega) over other bioluminescent reactions such as Gluc [19]; lower autoluminescence, brighter glow-type bioluminescent signal with >2-h half-life, enhanced physical/chemical stability, and improved expression in mammalian cells with little evidence of protein/antigen modifications.

We found that for T1D risk prediction, the ZnT8A RBA and LIPS assay had comparable performance. However, in comparison to other islet autoantibody markers measured by RBA (GADA/IA-2A/IAA ± ICA), Nluc-ZnT8 LIPS positivity further stratified T1D risk in relatives found positive for other islet autoantibodies that included rapid (<5 years) and slow (>10 years) progressors who developed diabetes. This confirmed previous findings that ZnT8A detection is useful in assessing diabetes risk in relatives positive for at least one other islet autoantibody, increasing the discrimination of high-risk individuals [5, 11, 13, 14]. A limitation of the present study is that the populations studied were pre-selected and therefore, we cannot predict how Nluc-ZnT8 LIPS would perform as an initial screening test. Other limitations include the high prevalence of diabetes in autoantibody-negative relatives as well as, the collection of diabetes diagnosis by questionnaire. However, the high prevalence of diabetes diagnosis can be largely explained by the increasing diagnosis of T2D with age-over-study follow-up (80.0% of ZnT8A negative relatives who progressed to diabetes reported a T2D diagnosis). Nevertheless, Nluc-ZnT8 LIPS was able to discriminate diabetes risk in both single and multiple autoantibody-positive relatives, which is highly representative of a T1D diabetes classification. Similar to many other studies in T1D, another limitation of the present study, is that by selecting autoantibody-positive relatives, individuals who may have developed ZnT8A at seroconversion, or those with single ZnT8A responses, would not be accounted for. Based on current literature, there is a low prevalence of single ZnT8A responses at diagnosis, and before diagnosis in relatives appears rare [1, 14]. They are also more likely to be associated with lower diabetes risk, like other single autoantibody responses [3, 21]. However, the prevalence of ZnT8A in populations of lower T1D genetic risk is not clear. There are also emerging data of additional epitopes other than the C-terminal of ZnT8, that may represent an earlier stage of ZnT8A autoimmunity, preceding GADA/IAA responses [34]. This requires further investigation.

While this study focused on T1D risk prediction in FDRs, emerging general population studies suggest that multiple autoantibodies are present in ~0.3% of subjects [35] versus ~3% in FDRs: data from >180 000 aged 2.5–45 years participating in TrialNet [36]. The difference in autoantibody prevalence has tremendous cost and feasibility implications for whole population screening of islet autoimmunity. For the last 20–30 years, RBAs have dominated islet autoantibody detection and remain one of the most commonly performed assays. Whilst the RBAs are low-volume and highly sensitive, RBAs are not only unsustainable in the long-term but they are also costly and cannot be readily adapted to the demands of population screening. Emerging immunoassays have sought to increase the viability of such studies. Among LIPS assays other examples include ELISA, electrochemiluminescence (ECL), and agglutination-PCR (ADAP). There have been LIPS, ELISA, and ECL methods described for ZnT8A detection, and when compared to RBA or ELISA, show comparable or improved assay performance [31, 37, 38]. All methods have inherent strengths and limitations that may benefit or restrict their use in research or clinical settings. A detailed discussion of these assays is beyond the scope of this manuscript, but automation of assays may be critical for large population screening.

Full automation is not possible for the Nluc-ZnT8 LIPS in its present format. A future avenue of this work would be to further adapt the assay to a plate-bound bridge-type ELISA for IgG/IgA/IgM detection, assess assay performance, and validate its compatibility in robotic systems. To achieve a plate-bound LIPS assay, large-scale ZnT8 protein production is needed which has been problematic, requiring stringent protocols for other immunoassay formats [39]. This could increase experimental costs but remains a key step for high-throughput assays. Integration of multiple antigens for simultaneous detection of multiple autoantibodies (multiplex) in either fluid- or plate-bound LIPS format is also viable, with the possibility of integrating different luciferases with distinct emission spectra to discriminate autoantibody specificity in a single test (e.g. Nluc with Firefly luciferase). Multiplex formats of ELISA, ECL, and ADAP assays in the context of T1D have been described [40–42], with ECL and ADAP able to distinguish autoantibody specificity.

The development of next-generation high-performing non-radioactive immunoassays, like this one, and licencing of immunotherapeutic agents [43, 44] in recent years, makes general population screening for early detection of at-risk individuals, reduced rates of individuals presenting with diabetic ketoacidosis at diagnosis, and primary/secondary intervention therapy to slow the rate of T1D progression an imminent reality.

## Supplementary Material

uxad139_suppl_Supplementary_Material

## Data Availability

The datasets are available from the corresponding author on reasonable request.

## Abbreviations

AS95: adjusted sensitivity at 95% specificity from ROC analysis
AU: arbitrary units
BOX: Bart’s-Oxford (BOX) family study
ELISA: enzyme-linked immunosorbent assay
FDR(s): first-degree relative(s)
Gluc: *Gaussia* luciferase
GADA: glutamic acid decarboxylase autoantibodies
IA-2A: islet antigen 2 autoantibodies
IAA: insulin autoantibodies
IASP: Islet Autoantibody Standardisation Program
LIPS: luciferase immunoprecipitation system
Nluc: Nano luciferase^TM^ (Promega, WI, USA)
Nluc-ZnT8: Nluc-ZnT8-R325 + ZnT8-W325 dual heterodimer
pROC-AUC(95): Partial ROC-AUC (specificity > 95%)
RBA: radiobinding assay
ROC-AUC: area under the receiver operator curve
T1D: type 1 diabetes
ZnT8A: zinc transporter 8 autoantibodies
ZnT8RA: zinc transporter R325 (arginine)-specific autoantibodies
ZnT8WA: zinc transporter W325 (tryptophan)-specific autoantibodies

## References

[1] Vermeulen I , Weets I, Asanghanwa M, Ruige J, Van Gaal L, Mathieu C, et al.; Belgian Diabetes Registry. Contribution of antibodies against IA-2beta and zinc transporter 8 to classification of diabetes diagnosed under 40 years of age. Diabetes Care 2011, 34, 1760–5. doi:10.2337/dc10-2268 21715527 PMC3142046

[2] Williams CL , Aitken RJ, Wilson IV, Mortimer GLM, Long AE, Williams AJK, et al.; BOX Study Group. The measurement of autoantibodies to insulin informs diagnosis of diabetes in a childhood population negative for other autoantibodies. Diabet Med 2022, 39, e14979. doi:10.1111/dme.14979 36251483 PMC9827938

[3] Ziegler AG , Rewers M, Simell O, Simell T, Lempainen J, Steck A, et al. Seroconversion to multiple islet autoantibodies and risk of progression to diabetes in children. JAMA 2013, 309, 2473–9. doi:10.1001/jama.2013.6285 23780460 PMC4878912

[4] Yu L , Rewers M, Gianani R, Kawasaki E, Zhang Y, Verge C, et al. Antiislet autoantibodies usually develop sequentially rather than simultaneously. J Clin Endocrinol Metab 1996, 81, 4264–7. doi:10.1210/jcem.81.12.8954025 8954025

[5] Gorus FK , Balti EV, Messaaoui A, Demeester S, Van Dalem A, Costa O, et al.; Belgian Diabetes Registry. Twenty-year progression rate to clinical onset according to autoantibody profile, age, and HLA-DQ genotype in a registry-based group of children and adults with a first-degree relative with type 1 diabetes. Diabetes Care 2017, 40, 1065–72. doi:10.2337/dc16-2228 28701370

[6] Achenbach P , Warncke K, Reiter J, Naserke HE, Williams AJ, Bingley PJ, et al. Stratification of type 1 diabetes risk on the basis of islet autoantibody characteristics. Diabetes 2004, 53, 384–92. doi:10.2337/diabetes.53.2.384 14747289

[7] Vehik K , Bonifacio E, Lernmark A, Yu L, Williams A, Schatz D, et al.; TEDDY Study Group. Hierarchical order of distinct autoantibody spreading and progression to type 1 diabetes in the TEDDY study. Diabetes Care 2020, 43, 2066–73. doi:10.2337/dc19-2547 32641373 PMC7440899

[8] Bingley PJ , Wherrett DK, Shultz A, Rafkin LE, Atkinson MA, Greenbaum CJ. Type 1 diabetes TrialNet: a multifaceted approach to bringing disease-modifying therapy to clinical use in type 1 diabetes. Diabetes Care 2018, 41, 653–61. doi:10.2337/dc17-0806 29559451 PMC5860837

[9] Long AE , Wilson IV, Becker DJ, Libman IM, Arena VC, Wong FS, et al. Characteristics of slow progression to diabetes in multiple islet autoantibody-positive individuals from five longitudinal cohorts: the SNAIL study. Diabetologia 2018, 61, 1484–90. doi:10.1007/s00125-018-4591-5 29532109 PMC6449004

[10] Wenzlau JM , Juhl K, Yu L, Moua O, Sarkar SA, Gottlieb P, et al. The cation efflux transporter ZnT8 (Slc30A8) is a major autoantigen in human type 1 diabetes. Proc Natl Acad Sci U S A 2007, 104, 17040–5. doi:10.1073/pnas.0705894104 17942684 PMC2040407

[11] Achenbach P , Lampasona V, Landherr U, Koczwara K, Krause S, Grallert H, et al. Autoantibodies to zinc transporter 8 and SLC30A8 genotype stratify type 1 diabetes risk. Diabetologia 2009, 52, 1881–8. doi:10.1007/s00125-009-1438-0 19590848

[12] Ziegler AG , Bonifacio E, Group B-BS. Age-related islet autoantibody incidence in offspring of patients with type 1 diabetes. Diabetologia 2012, 55, 1937–43. doi: 10.1007/s00125-012-2472-x 22289814

[13] De Grijse J , Asanghanwa M, Nouthe B, Albrecher N, Goubert P, Vermeulen I, et al.; Belgian Diabetes Registry. Predictive power of screening for antibodies against insulinoma-associated protein 2 beta (IA-2beta) and zinc transporter-8 to select first-degree relatives of type 1 diabetic patients with risk of rapid progression to clinical onset of the disease: implications for prevention trials. Diabetologia 2010, 53, 517–24. doi:10.1007/s00125-009-1618-y 20091020

[14] Yu L , Boulware DC, Beam CA, Hutton JC, Wenzlau JM, Greenbaum CJ, et al.; Type 1 Diabetes TrialNet Study Group. Zinc transporter-8 autoantibodies improve prediction of type 1 diabetes in relatives positive for the standard biochemical autoantibodies. Diabetes Care 2012, 35, 1213–8. doi:10.2337/dc11-2081 22446173 PMC3357246

[15] Gorus FK , Balti EV, Vermeulen I, Demeester S, Van Dalem A, Costa O, et al.; Belgian Diabetes Registry. Screening for insulinoma antigen 2 and zinc transporter 8 autoantibodies: a cost-effective and age-independent strategy to identify rapid progressors to clinical onset among relatives of type 1 diabetic patients. Clin Exp Immunol 2013, 171, 82–90. doi:10.1111/j.1365-2249.2012.04675.x 23199327 PMC3530099

[16] Wenzlau JM , Liu Y, Yu L, Moua O, Fowler KT, Rangasamy S, et al. A common nonsynonymous single nucleotide polymorphism in the SLC30A8 gene determines ZnT8 autoantibody specificity in type 1 diabetes. Diabetes 2008, 57, 2693–7. doi:10.2337/db08-0522 18591387 PMC2551679

[17] The Eurodiab Ace Study Group. Familial risk of type I diabetes in European children The Eurodiab Ace Study Group and The Eurodiab Ace Substudy 2 Study Group. Diabetologia 1998, 41, 1151–6. doi:10.1007/s001250051044 9794100

[18] Liberati D , Wyatt RC, Brigatti C, Marzinotto I, Ferrari M, Bazzigaluppi E, et al. A novel LIPS assay for insulin autoantibodies. Acta Diabetol 2018, 55, 263–70. doi:10.1007/s00592-017-1082-y 29305766

[19] Hall MP , Unch J, Binkowski BF, Valley MP, Butler BL, Wood MG, et al. Engineered luciferase reporter from a deep sea shrimp utilizing a novel imidazopyrazinone substrate. ACS Chem Biol 2012, 7, 1848–57. doi:10.1021/cb3002478 22894855 PMC3501149

[20] Gillespie KM , Fareed R, Mortimer GL. Four decades of the Bart’s Oxford study: Improved tests to predict type 1 diabetes. Diabet Med 2021, 38, e14717. doi:10.1111/dme.14717 34655243

[21] Bingley PJ , Gale EA; European Nicotinamide Diabetes Intervention Trial (ENDIT) Group. Progression to type 1 diabetes in islet cell antibody-positive relatives in the European Nicotinamide Diabetes Intervention Trial: the role of additional immune, genetic and metabolic markers of risk. Diabetologia 2006, 49, 881–90. doi:10.1007/s00125-006-0160-4 16514546

[22] Bingley PJ , Bonifacio E, Williams AJ, Genovese S, Bottazzo GF, Gale EA. Prediction of IDDM in the general population: strategies based on combinations of autoantibody markers. Diabetes 1997, 46, 1701–10. doi:10.2337/diab.46.11.1701 9356015

[23] Bonifacio E , Yu L, Williams AK, Eisenbarth GS, Bingley PJ, Marcovina SM, et al. Harmonization of glutamic acid decarboxylase and islet antigen-2 autoantibody assays for National Institute of Diabetes and digestive and kidney diseases consortia. J Clin Endocrinol Metab 2010, 95, 3360–7. doi:10.1210/jc.2010-0293 20444913 PMC2928900

[24] Williams AJ , Bingley PJ, Bonifacio E, Palmer JP, Gale EA. A novel micro-assay for insulin autoantibodies. J Autoimmun 1997, 10, 473–8. doi:10.1006/jaut.1997.0154 9376075

[25] Bingley PJ , Bonifacio E, Shattock M, Gillmor HA, Sawtell PA, Dunger DB, et al. Can islet cell antibodies predict IDDM in the general population? Diabetes Care 1993, 16, 45–50. doi:10.2337/diacare.16.1.45 8422831

[26] Long AE , Gillespie KM, Rokni S, Bingley PJ, Williams AJ. Rising incidence of type 1 diabetes is associated with altered immunophenotype at diagnosis. Diabetes 2012, 61, 683–6. doi:10.2337/db11-0962 22315309 PMC3282823

[27] Wyatt RC , Grace SL, Brigatti C, Liberati D, Gillard BT, Marzinotto I, et al. Improved specificity of glutamate decarboxylase 65 autoantibody measurement using luciferase-based immunoprecipitation system (LIPS) assays. medRxiv 2023. doi:10.1101/2023.07.03.23292157. PMC10958581 38232306

[28] Robin X , Turck N, Hainard A, Tiberti N, Lisacek F, Sanchez JC, et al. pROC: an open-source package for R and S+ to analyze and compare ROC curves. BMC Bioinf 2011, 12, 77. doi:10.1186/1471-2105-12-77 PMC3068975 21414208

[29] Marcus P , Yan X, Bartley B, Hagopian W. LIPS islet autoantibody assays in high-throughput format for DASP 2010. Diabetes Metab Res Rev 2011, 27, 891–4. doi:10.1002/dmrr.1268 22069280

[30] Burbelo PD , Hirai H, Leahy H, Lernmark A, Ivarsson SA, Iadarola MJ, et al. A new luminescence assay for autoantibodies to mammalian cell-prepared insulinoma-associated protein 2. Diabetes Care 2008, 31, 1824–6. doi:10.2337/dc08-0286 18535195 PMC2518352

[31] Ustinova J , Zusinaite E, Utt M, Metskula K, Reimand K, Huchaiah V, et al. Development of a luciferase-based system for the detection of ZnT8 autoantibodies. J Immunol Methods 2014, 405, 67–73. doi:10.1016/j.jim.2014.01.009 24462542

[32] McLaughlin KA , Richardson CC, Ravishankar A, Brigatti C, Liberati D, Lampasona V, et al. Identification of tetraspanin-7 as a target of autoantibodies in type 1 diabetes. Diabetes 2016, 65, 1690–8. doi:10.2337/db15-1058 26953162

[33] Grace SL , Cooper A, Jones AG, McDonald TJ. Zinc transporter 8 autoantibody testing requires age-related cut-offs. BMJ Open Diabetes Res Care 2021, 9, e002296. doi:10.1136/bmjdrc-2021-002296 PMC8340275 34348918

[34] Gu Y , Merriman C, Guo Z, Jia X, Wenzlau J, Li H, et al. Novel autoantibodies to the beta-cell surface epitopes of ZnT8 in patients progressing to type-1 diabetes. J Autoimmun 2021, 122, 102677. doi:10.1016/j.jaut.2021.102677 34130115 PMC9029399

[35] Ziegler AG , Kick K, Bonifacio E, Haupt F, Hippich M, Dunstheimer D, et al.; Fr1da Study Group. Yield of a public health screening of children for islet autoantibodies in Bavaria, Germany. JAMA 2020, 323, 339–51. doi:10.1001/jama.2019.21565 31990315 PMC6990943

[36] Sims EK , Geyer S, Johnson SB, Libman I, Jacobsen LM, Boulware D, et al.; Type 1 Diabetes TrialNet Study Group. Who is enrolling? The path to monitoring in type 1 diabetes TrialNet’s pathway to prevention. Diabetes Care 2019, 42, 2228–36. doi:10.2337/dc19-0593 31558546 PMC6868467

[37] Dunseath G , Ananieva-Jordanova R, Coles R, Powell M, Amoroso M, Furmaniak J, et al. Bridging-type enzyme-linked immunoassay for zinc transporter 8 autoantibody measurements in adult patients with diabetes mellitus. Clin Chim Acta 2015, 447, 90–5. doi:10.1016/j.cca.2015.05.010 26006309

[38] Jia X , He L, Miao D, Waugh K, Geno Rasmussen C, Dong F, et al. High-affinity ZnT8 autoantibodies by electrochemiluminescence assay improve risk prediction for type 1 diabetes. J Clin Endocrinol Metab 2021, 106, 3455–3463. 34343303 10.1210/clinem/dgab575 PMC8864749

[39] Wan H , Merriman C, Atkinson MA, Wasserfall CH, McGrail KM, Liang Y, et al. Proteoliposome-based full-length ZnT8 self-antigen for type 1 diabetes diagnosis on a plasmonic platform. Proc Natl Acad Sci U S A 2017, 114, 10196–201. doi:10.1073/pnas.1711169114 28874568 PMC5617307

[40] Amoroso M , Achenbach P, Powell M, Coles R, Chlebowska M, Carr L, et al. 3 Screen islet cell autoantibody ELISA: a sensitive and specific ELISA for the combined measurement of autoantibodies to GAD65, to IA-2 and to ZnT8. Clin Chim Acta 2016, 462, 60–4. doi:10.1016/j.cca.2016.08.013 27570064

[41] He L , Jia X, Rasmussen CG, Waugh K, Miao D, Dong F, et al. High-throughput multiplex electrochemiluminescence assay applicable to general population screening for type 1 diabetes and celiac disease. Diabetes Technol Ther 2022, 24, 502–9. doi:10.1089/dia.2021.0517 35238620 PMC9464081

[42] Lind A , de Jesus Cortez F, Ramelius A, Bennet R, Robinson PV, Seftel D, et al. Multiplex agglutination-PCR (ADAP) autoantibody assays compared to radiobinding autoantibodies in type 1 diabetes and celiac disease. J Immunol Methods 2022, 506, 113265. doi:10.1016/j.jim.2022.113265 35358496

[43] Herold KC , Bundy BN, Long SA, Bluestone JA, DiMeglio LA, Dufort MJ, et al.; Type 1 Diabetes TrialNet Study Group. An anti-CD3 antibody, teplizumab, in relatives at risk for type 1 diabetes. N Engl J Med 2019, 381, 603–13. doi:10.1056/NEJMoa1902226 31180194 PMC6776880

[44] Sims EK , Bundy BN, Stier K, Serti E, Lim N, Long SA, et al. Teplizumab improves and stabilizes beta cell function in antibody-positive high-risk individuals. Sci Transl Med 2021, 13, 1–14. doi:10.1126/scitranslmed.abc8980 PMC8610022 33658358

