# Supplementary material for: A novel, high-performance, low-volume, rapid luciferase immunoprecipitation system (LIPS) assay to detect autoantibodies to zinc transporter 8
Source: Clin Exp Immunol. 2023 Dec 27;215(3):215–24. doi: 10.1093/cei/uxad139 (PMC10876106; doi:10.1093/cei/uxad139)
Supplement: uxad139_suppl_Supplementary_Material [file uxad139_suppl_supplementary_material.pptx]

## Slide 1
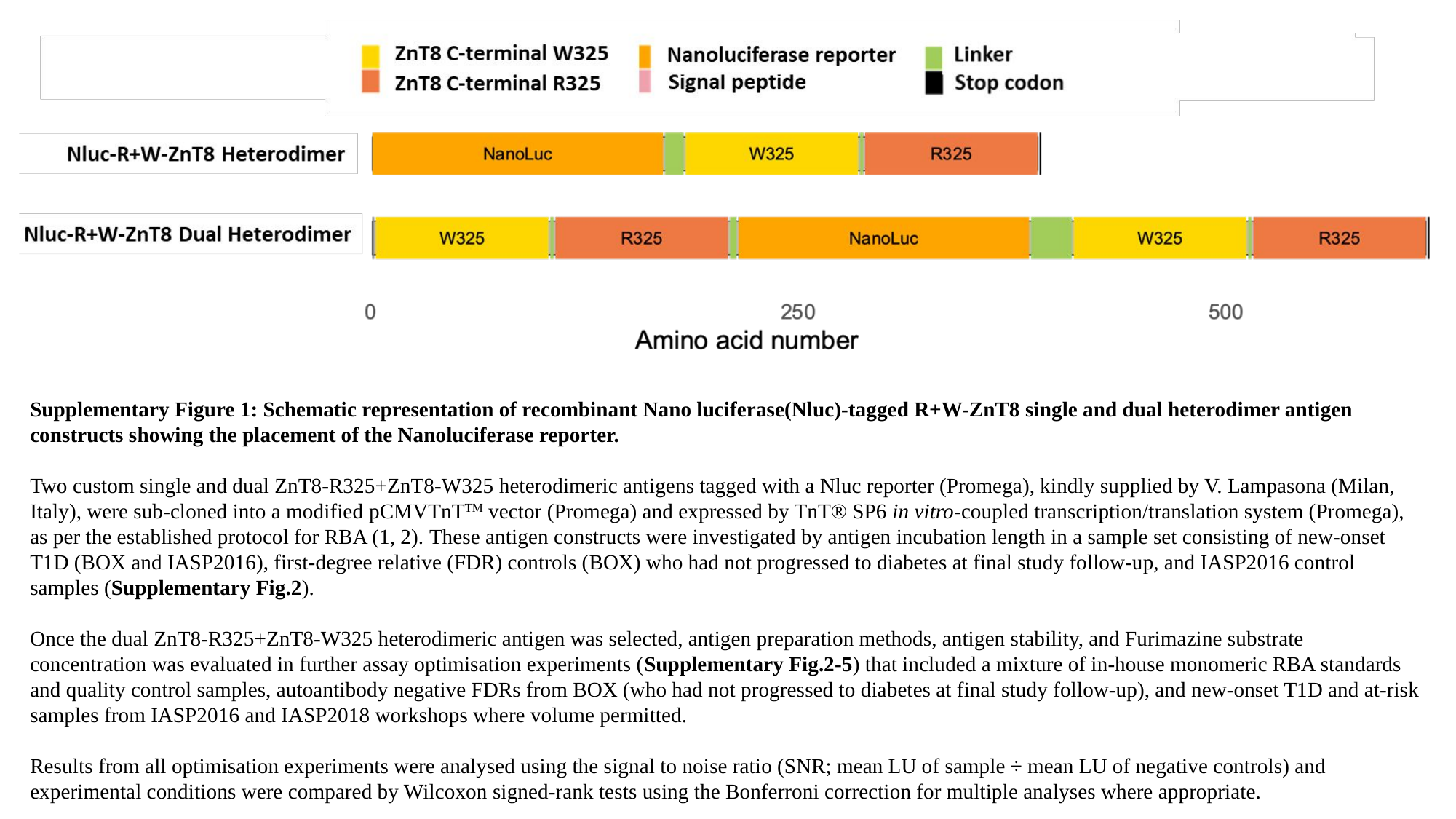

Supplementary Figure 1: Schematic representation of recombinant Nano luciferase(Nluc)-tagged R+W-ZnT8 single and dual heterodimer antigen constructs showing the placement of the Nanoluciferase reporter.
Two custom single and dual ZnT8-R325+ZnT8-W325 heterodimeric antigens tagged with a Nluc reporter (Promega), kindly supplied by V. Lampasona (Milan, Italy), were sub-cloned into a modified pCMVTnTTM vector (Promega) and expressed by TnT® SP6 in vitro-coupled transcription/translation system (Promega), as per the established protocol for RBA (1, 2). These antigen constructs were investigated by antigen incubation length in a sample set consisting of new-onset T1D (BOX and IASP2016), first-degree relative (FDR) controls (BOX) who had not progressed to diabetes at final study follow-up, and IASP2016 control samples (Supplementary Fig.2).
Once the dual ZnT8-R325+ZnT8-W325 heterodimeric antigen was selected, antigen preparation methods, antigen stability, and Furimazine substrate concentration was evaluated in further assay optimisation experiments (Supplementary Fig.2-5) that included a mixture of in-house monomeric RBA standards and quality control samples, autoantibody negative FDRs from BOX (who had not progressed to diabetes at final study follow-up), and new-onset T1D and at-risk samples from IASP2016 and IASP2018 workshops where volume permitted.
Results from all optimisation experiments were analysed using the signal to noise ratio (SNR; mean LU of sample ÷ mean LU of negative controls) and experimental conditions were compared by Wilcoxon signed-rank tests using the Bonferroni correction for multiple analyses where appropriate.

## Slide 2
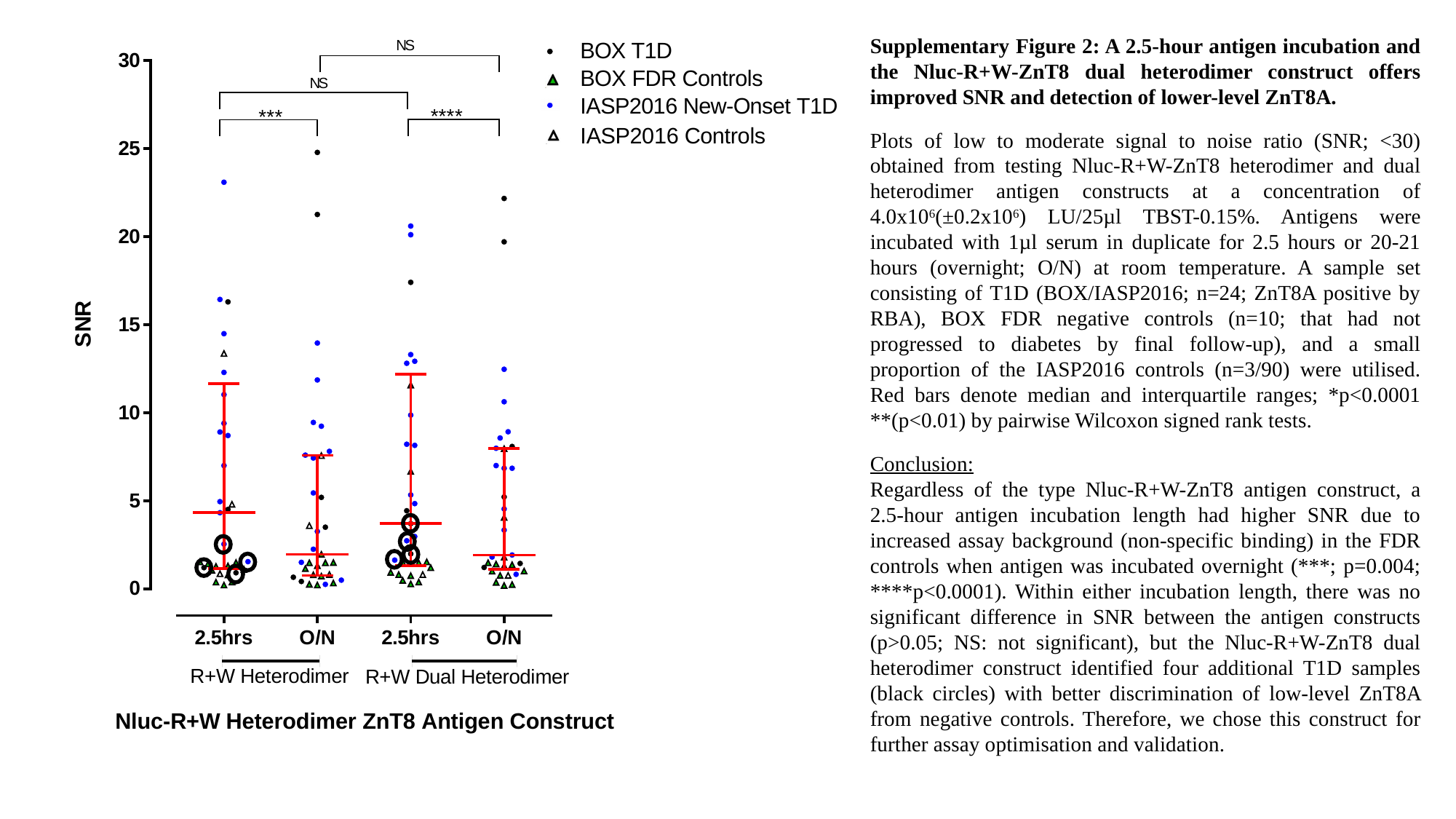

Supplementary Figure 2: A 2.5-hour antigen incubation and the Nluc-R+W-ZnT8 dual heterodimer construct offers improved SNR and detection of lower-level ZnT8A.
Plots of low to moderate signal to noise ratio (SNR; <30) obtained from testing Nluc-R+W-ZnT8 heterodimer and dual heterodimer antigen constructs at a concentration of 4.0x10^6^(±0.2x10^6^) LU/25µl TBST-0.15%. Antigens were incubated with 1µl serum in duplicate for 2.5 hours or 20-21 hours (overnight; O/N) at room temperature. A sample set consisting of T1D (BOX/IASP2016; n=24; ZnT8A positive by RBA), BOX FDR negative controls (n=10; that had not progressed to diabetes by final follow-up), and a small proportion of the IASP2016 controls (n=3/90) were utilised. Red bars denote median and interquartile ranges; *p<0.0001 **(p<0.01) by pairwise Wilcoxon signed rank tests.
Conclusion:
Regardless of the type Nluc-R+W-ZnT8 antigen construct, a 2.5-hour antigen incubation length had higher SNR due to increased assay background (non-specific binding) in the FDR controls when antigen was incubated overnight (***; p=0.004; ****p<0.0001). Within either incubation length, there was no significant difference in SNR between the antigen constructs (p>0.05; NS: not significant), but the Nluc-R+W-ZnT8 dual heterodimer construct identified four additional T1D samples (black circles) with better discrimination of low-level ZnT8A from negative controls. Therefore, we chose this construct for further assay optimisation and validation.

## Slide 3
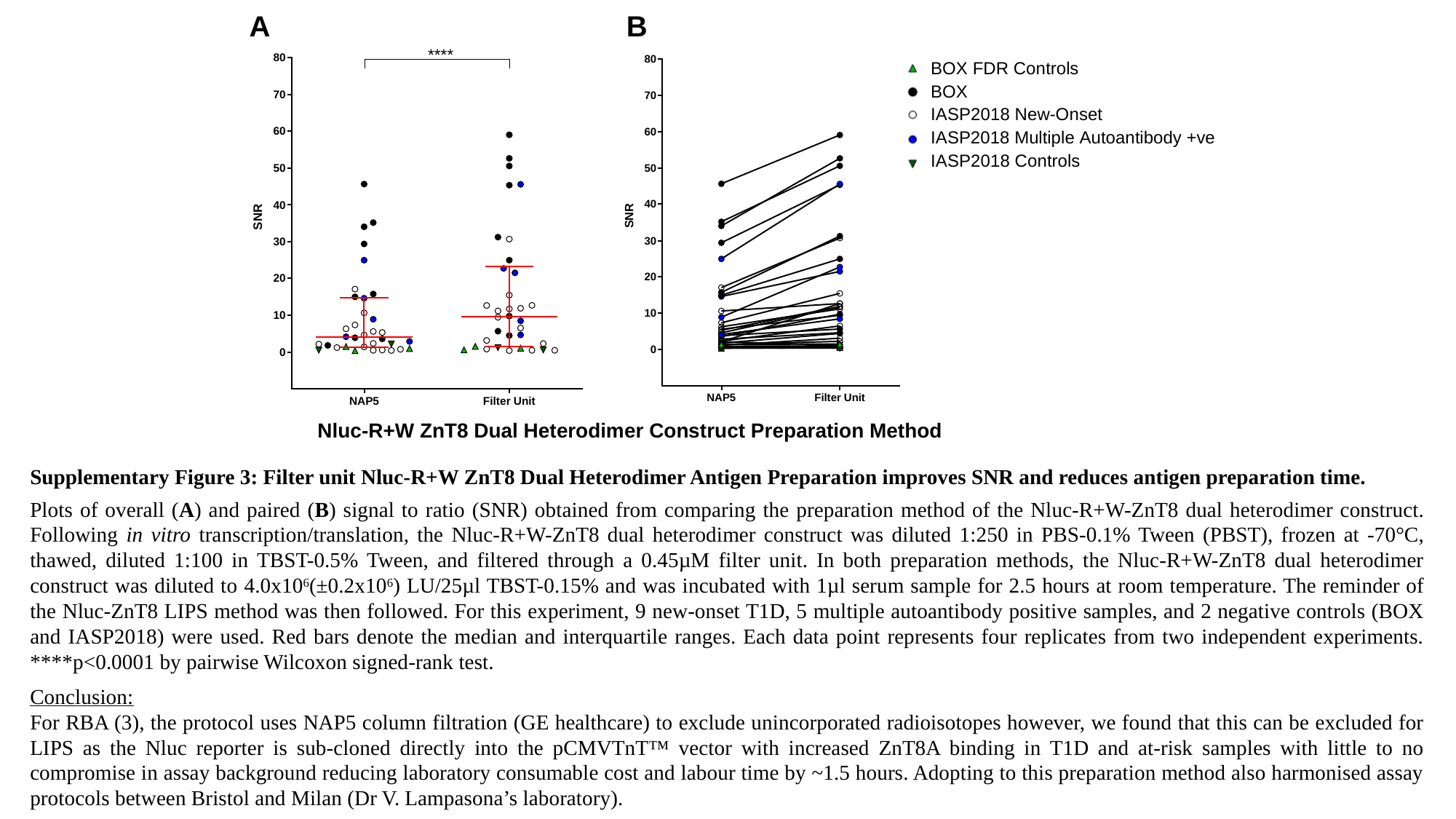

Supplementary Figure 3: Filter unit Nluc-R+W ZnT8 Dual Heterodimer Antigen Preparation improves SNR and reduces antigen preparation time.
Plots of overall (A) and paired (B) signal to ratio (SNR) obtained from comparing the preparation method of the Nluc-R+W-ZnT8 dual heterodimer construct. Following in vitro transcription/translation, the Nluc-R+W-ZnT8 dual heterodimer construct was diluted 1:250 in PBS-0.1% Tween (PBST), frozen at -70°C, thawed, diluted 1:100 in TBST-0.5% Tween, and filtered through a 0.45µM filter unit. In both preparation methods, the Nluc-R+W-ZnT8 dual heterodimer construct was diluted to 4.0x10^6^(±0.2x10^6^) LU/25µl TBST-0.15% and was incubated with 1µl serum sample for 2.5 hours at room temperature. The reminder of the Nluc-ZnT8 LIPS method was then followed. For this experiment, 9 new-onset T1D, 5 multiple autoantibody positive samples, and 2 negative controls (BOX and IASP2018) were used. Red bars denote the median and interquartile ranges. Each data point represents four replicates from two independent experiments. ****p<0.0001 by pairwise Wilcoxon signed-rank test.
Conclusion:
For RBA (3), the protocol uses NAP5 column filtration (GE healthcare) to exclude unincorporated radioisotopes however, we found that this can be excluded for LIPS as the Nluc reporter is sub-cloned directly into the pCMVTnT™ vector with increased ZnT8A binding in T1D and at-risk samples with little to no compromise in assay background reducing laboratory consumable cost and labour time by ~1.5 hours. Adopting to this preparation method also harmonised assay protocols between Bristol and Milan (Dr V. Lampasona’s laboratory).

## Slide 4
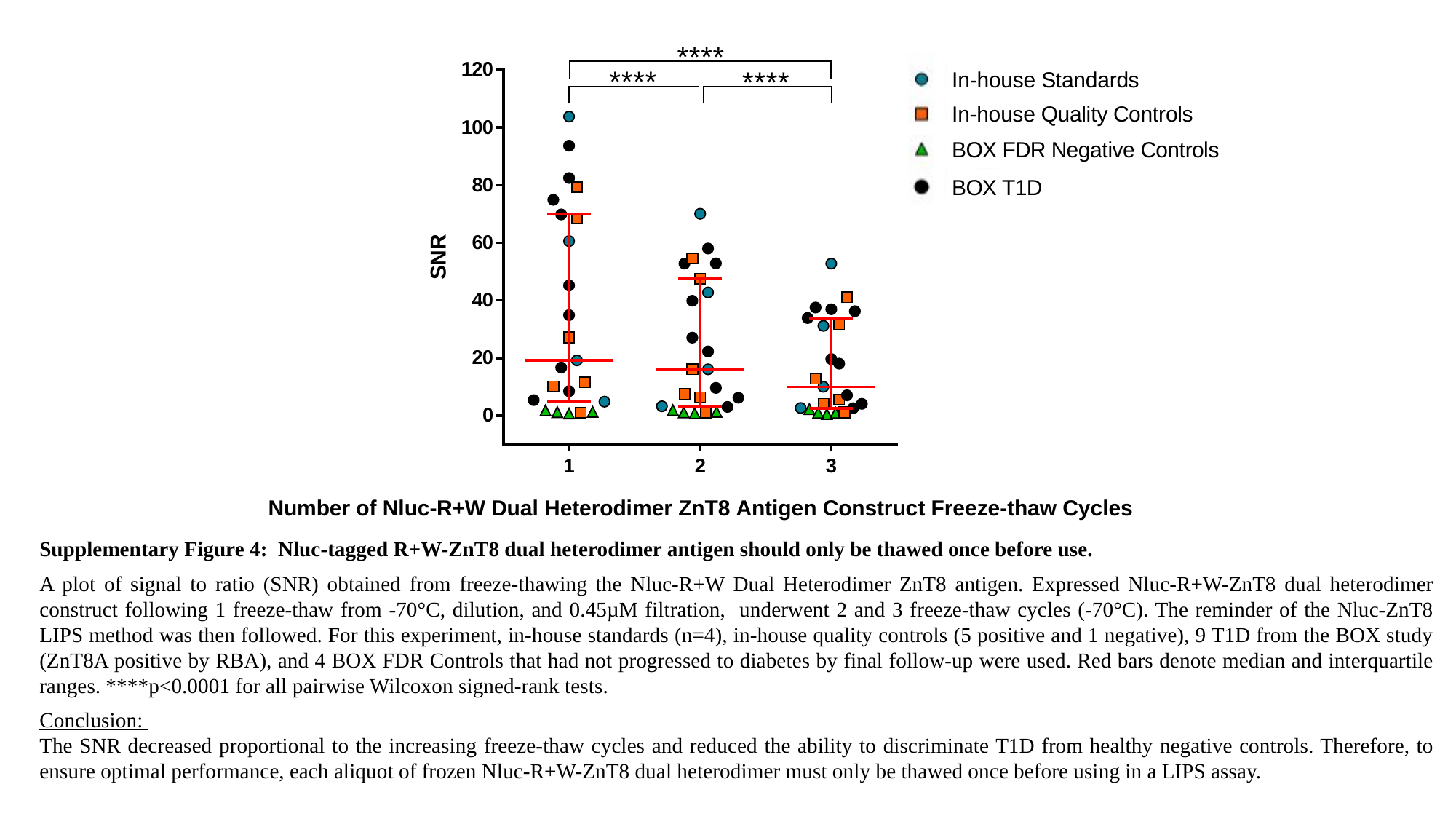

Supplementary Figure 4: Nluc-tagged R+W-ZnT8 dual heterodimer antigen should only be thawed once before use.
A plot of signal to ratio (SNR) obtained from freeze-thawing the Nluc-R+W Dual Heterodimer ZnT8 antigen. Expressed Nluc-R+W-ZnT8 dual heterodimer construct following 1 freeze-thaw from -70°C, dilution, and 0.45µM filtration, underwent 2 and 3 freeze-thaw cycles (-70°C). The reminder of the Nluc-ZnT8 LIPS method was then followed. For this experiment, in-house standards (n=4), in-house quality controls (5 positive and 1 negative), 9 T1D from the BOX study (ZnT8A positive by RBA), and 4 BOX FDR Controls that had not progressed to diabetes by final follow-up were used. Red bars denote median and interquartile ranges. ****p<0.0001 for all pairwise Wilcoxon signed-rank tests.
Conclusion:
The SNR decreased proportional to the increasing freeze-thaw cycles and reduced the ability to discriminate T1D from healthy negative controls. Therefore, to ensure optimal performance, each aliquot of frozen Nluc-R+W-ZnT8 dual heterodimer must only be thawed once before using in a LIPS assay.

## Slide 5
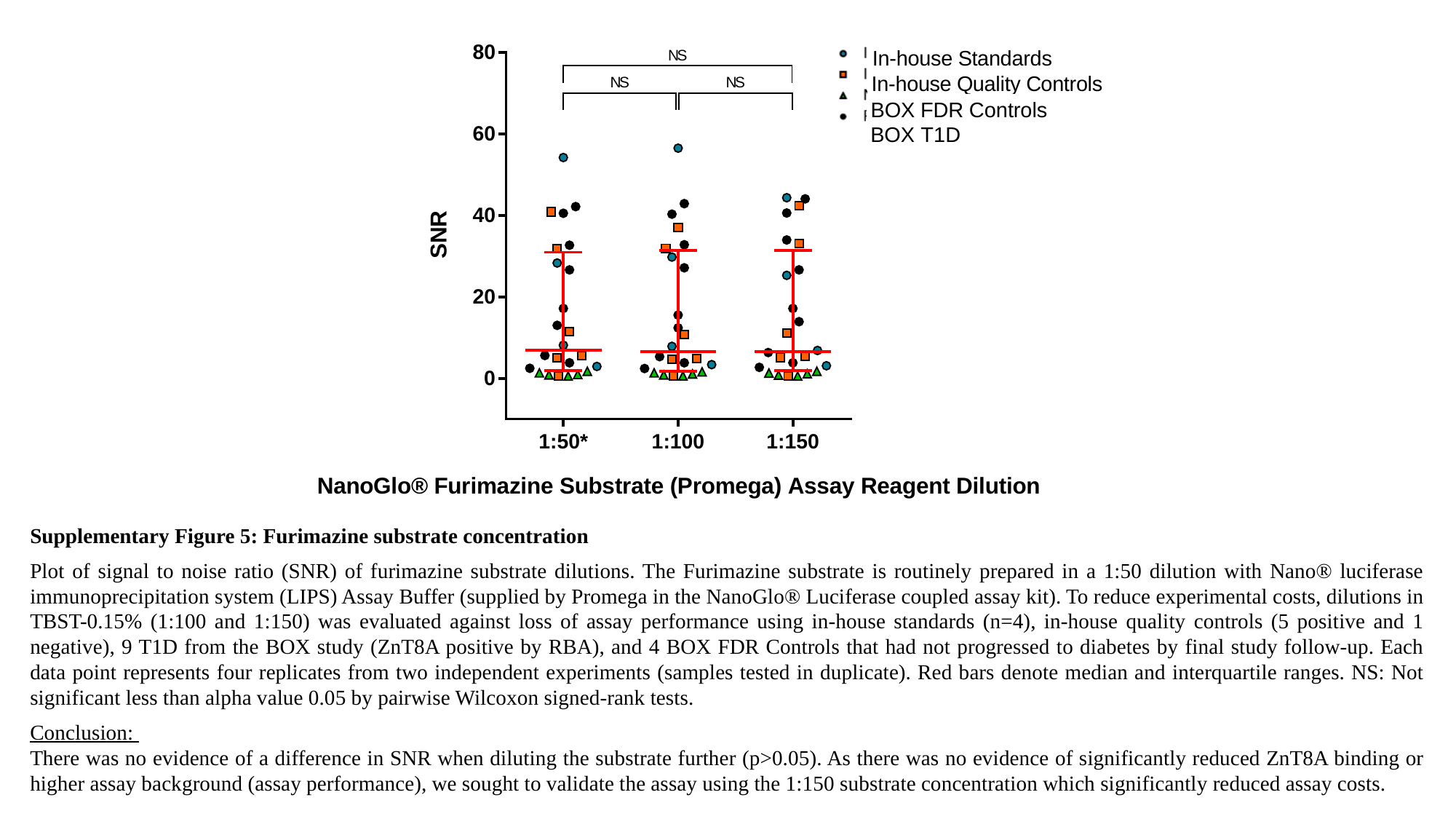

Supplementary Figure 5: Furimazine substrate concentration
Plot of signal to noise ratio (SNR) of furimazine substrate dilutions. The Furimazine substrate is routinely prepared in a 1:50 dilution with Nano® luciferase immunoprecipitation system (LIPS) Assay Buffer (supplied by Promega in the NanoGlo® Luciferase coupled assay kit). To reduce experimental costs, dilutions in TBST-0.15% (1:100 and 1:150) was evaluated against loss of assay performance using in-house standards (n=4), in-house quality controls (5 positive and 1 negative), 9 T1D from the BOX study (ZnT8A positive by RBA), and 4 BOX FDR Controls that had not progressed to diabetes by final study follow-up. Each data point represents four replicates from two independent experiments (samples tested in duplicate). Red bars denote median and interquartile ranges. NS: Not significant less than alpha value 0.05 by pairwise Wilcoxon signed-rank tests.
Conclusion:
There was no evidence of a difference in SNR when diluting the substrate further (p>0.05). As there was no evidence of significantly reduced ZnT8A binding or higher assay background (assay performance), we sought to validate the assay using the 1:150 substrate concentration which significantly reduced assay costs.

## Slide 6
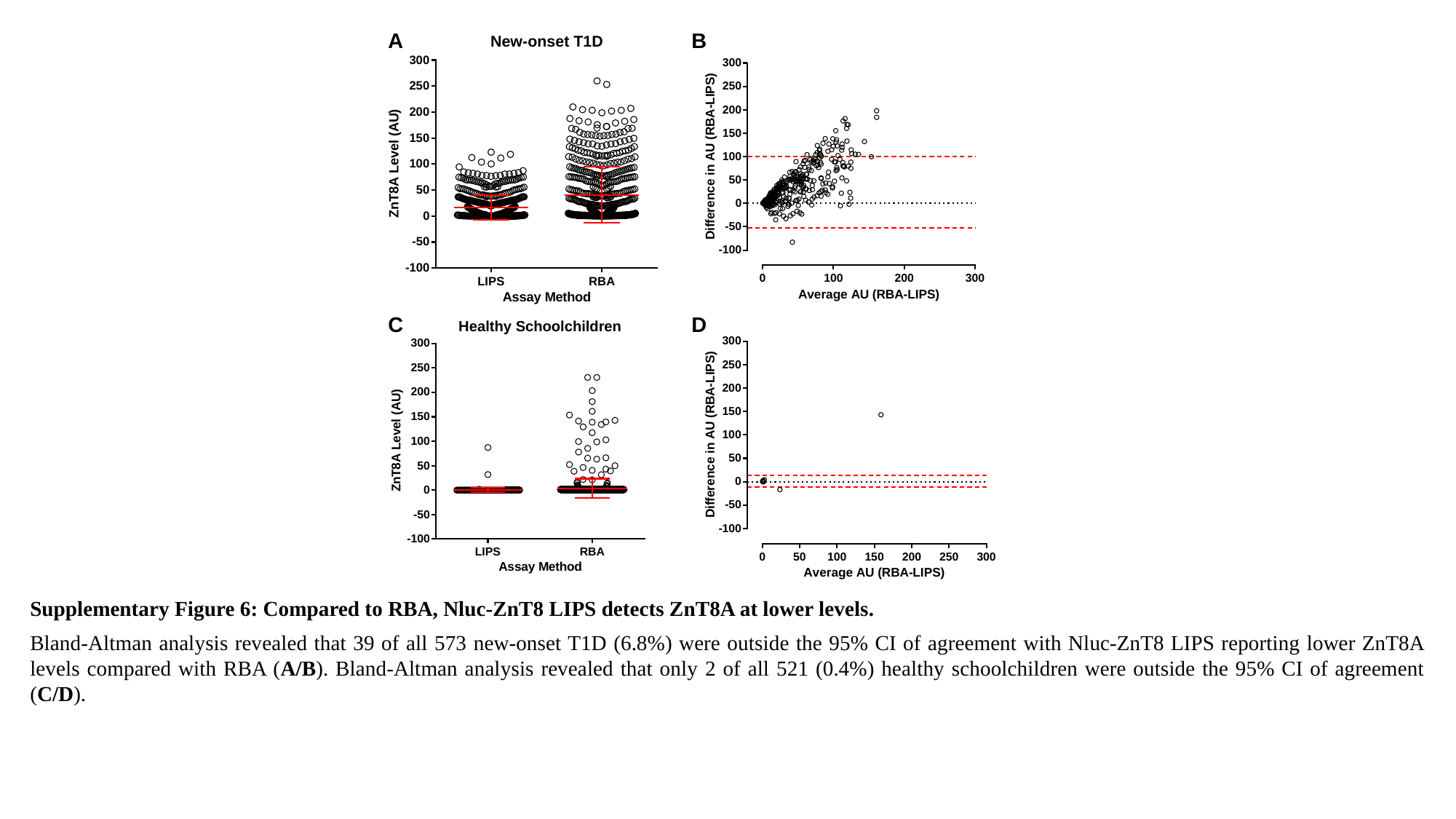

Supplementary Figure 6: Compared to RBA, Nluc-ZnT8 LIPS detects ZnT8A at lower levels.
Bland-Altman analysis revealed that 39 of all 573 new-onset T1D (6.8%) were outside the 95% CI of agreement with Nluc-ZnT8 LIPS reporting lower ZnT8A levels compared with RBA (A/B). Bland-Altman analysis revealed that only 2 of all 521 (0.4%) healthy schoolchildren were outside the 95% CI of agreement (C/D).

## Slide 7
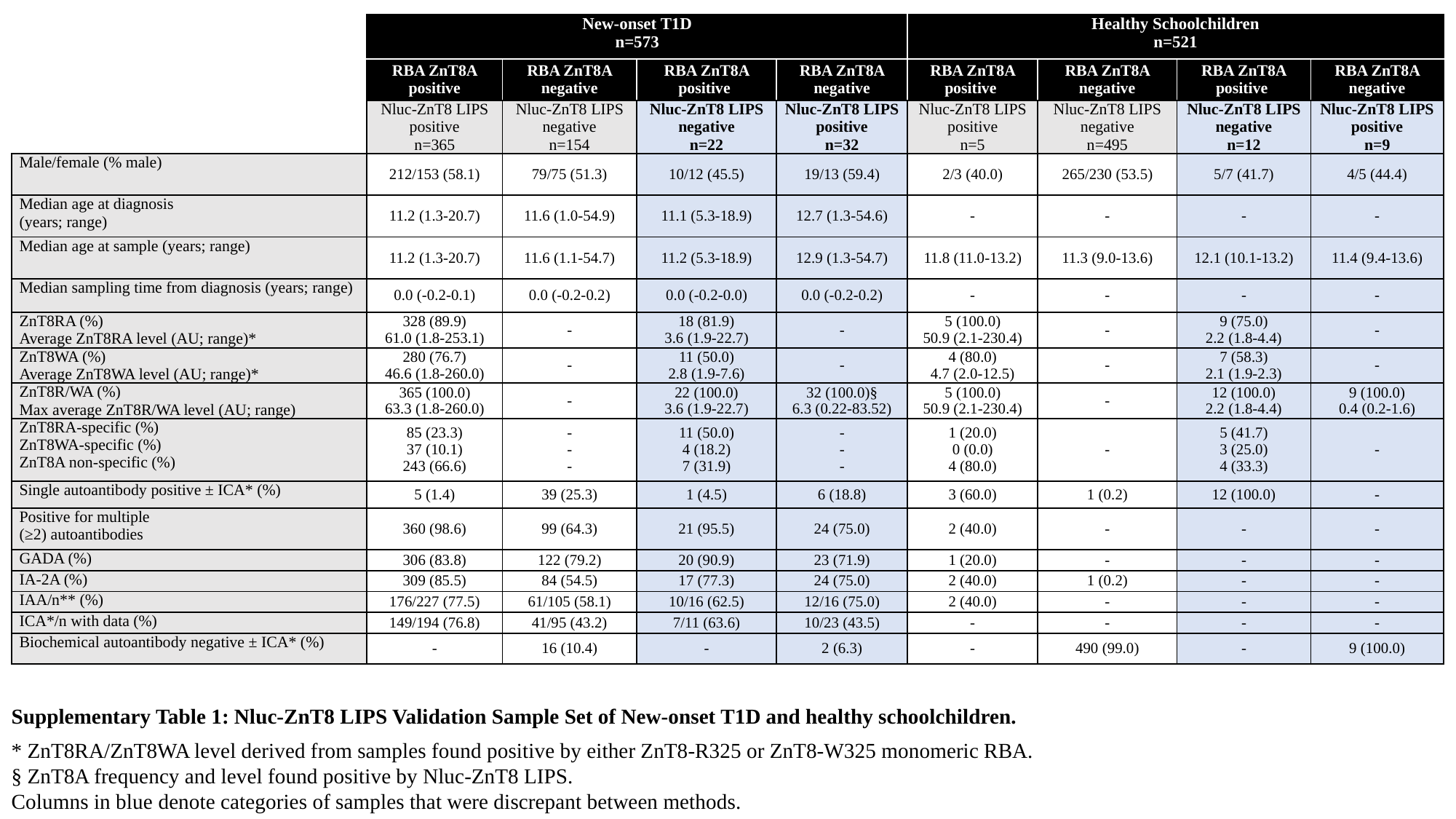

| | New-onset T1D n=573 | | | | Healthy Schoolchildren n=521 | | | |
| --- | --- | --- | --- | --- | --- | --- | --- | --- |
| | RBA ZnT8A positive | RBA ZnT8A negative | RBA ZnT8A positive | RBA ZnT8A negative | RBA ZnT8A positive | RBA ZnT8A negative | RBA ZnT8A positive | RBA ZnT8A negative |
| | Nluc-ZnT8 LIPS positive n=365 | Nluc-ZnT8 LIPS negative n=154 | Nluc-ZnT8 LIPS negative n=22 | Nluc-ZnT8 LIPS positive n=32 | Nluc-ZnT8 LIPS positive n=5 | Nluc-ZnT8 LIPS negative n=495 | Nluc-ZnT8 LIPS negative n=12 | Nluc-ZnT8 LIPS positive n=9 |
| Male/female (% male) | 212/153 (58.1) | 79/75 (51.3) | 10/12 (45.5) | 19/13 (59.4) | 2/3 (40.0) | 265/230 (53.5) | 5/7 (41.7) | 4/5 (44.4) |
| Median age at diagnosis (years; range) | 11.2 (1.3-20.7) | 11.6 (1.0-54.9) | 11.1 (5.3-18.9) | 12.7 (1.3-54.6) | - | - | - | - |
| Median age at sample (years; range) | 11.2 (1.3-20.7) | 11.6 (1.1-54.7) | 11.2 (5.3-18.9) | 12.9 (1.3-54.7) | 11.8 (11.0-13.2) | 11.3 (9.0-13.6) | 12.1 (10.1-13.2) | 11.4 (9.4-13.6) |
| Median sampling time from diagnosis (years; range) | 0.0 (-0.2-0.1) | 0.0 (-0.2-0.2) | 0.0 (-0.2-0.0) | 0.0 (-0.2-0.2) | - | - | - | - |
| ZnT8RA (%) Average ZnT8RA level (AU; range)\* | 328 (89.9) 61.0 (1.8-253.1) | - | 18 (81.9) 3.6 (1.9-22.7) | - | 5 (100.0) 50.9 (2.1-230.4) | - | 9 (75.0) 2.2 (1.8-4.4) | - |
| ZnT8WA (%) Average ZnT8WA level (AU; range)\* | 280 (76.7) 46.6 (1.8-260.0) | - | 11 (50.0) 2.8 (1.9-7.6) | - | 4 (80.0) 4.7 (2.0-12.5) | - | 7 (58.3) 2.1 (1.9-2.3) | - |
| ZnT8R/WA (%) Max average ZnT8R/WA level (AU; range) | 365 (100.0) 63.3 (1.8-260.0) | - | 22 (100.0) 3.6 (1.9-22.7) | 32 (100.0)§ 6.3 (0.22-83.52) | 5 (100.0) 50.9 (2.1-230.4) | - | 12 (100.0) 2.2 (1.8-4.4) | 9 (100.0) 0.4 (0.2-1.6) |
| ZnT8RA-specific (%) ZnT8WA-specific (%) ZnT8A non-specific (%) | 85 (23.3) 37 (10.1) 243 (66.6) | - - - | 11 (50.0) 4 (18.2) 7 (31.9) | - - - | 1 (20.0) 0 (0.0) 4 (80.0) | - | 5 (41.7) 3 (25.0) 4 (33.3) | - |
| Single autoantibody positive ± ICA\* (%) | 5 (1.4) | 39 (25.3) | 1 (4.5) | 6 (18.8) | 3 (60.0) | 1 (0.2) | 12 (100.0) | - |
| Positive for multiple (≥2) autoantibodies | 360 (98.6) | 99 (64.3) | 21 (95.5) | 24 (75.0) | 2 (40.0) | - | - | - |
| GADA (%) | 306 (83.8) | 122 (79.2) | 20 (90.9) | 23 (71.9) | 1 (20.0) | - | - | - |
| IA-2A (%) | 309 (85.5) | 84 (54.5) | 17 (77.3) | 24 (75.0) | 2 (40.0) | 1 (0.2) | - | - |
| IAA/n\*\* (%) | 176/227 (77.5) | 61/105 (58.1) | 10/16 (62.5) | 12/16 (75.0) | 2 (40.0) | - | - | - |
| ICA\*/n with data (%) | 149/194 (76.8) | 41/95 (43.2) | 7/11 (63.6) | 10/23 (43.5) | - | - | - | - |
| Biochemical autoantibody negative ± ICA\* (%) | - | 16 (10.4) | - | 2 (6.3) | - | 490 (99.0) | - | 9 (100.0) |
Supplementary Table 1: Nluc-ZnT8 LIPS Validation Sample Set of New-onset T1D and healthy schoolchildren.
* ZnT8RA/ZnT8WA level derived from samples found positive by either ZnT8-R325 or ZnT8-W325 monomeric RBA.
§ ZnT8A frequency and level found positive by Nluc-ZnT8 LIPS.
Columns in blue denote categories of samples that were discrepant between methods.

## Slide 8
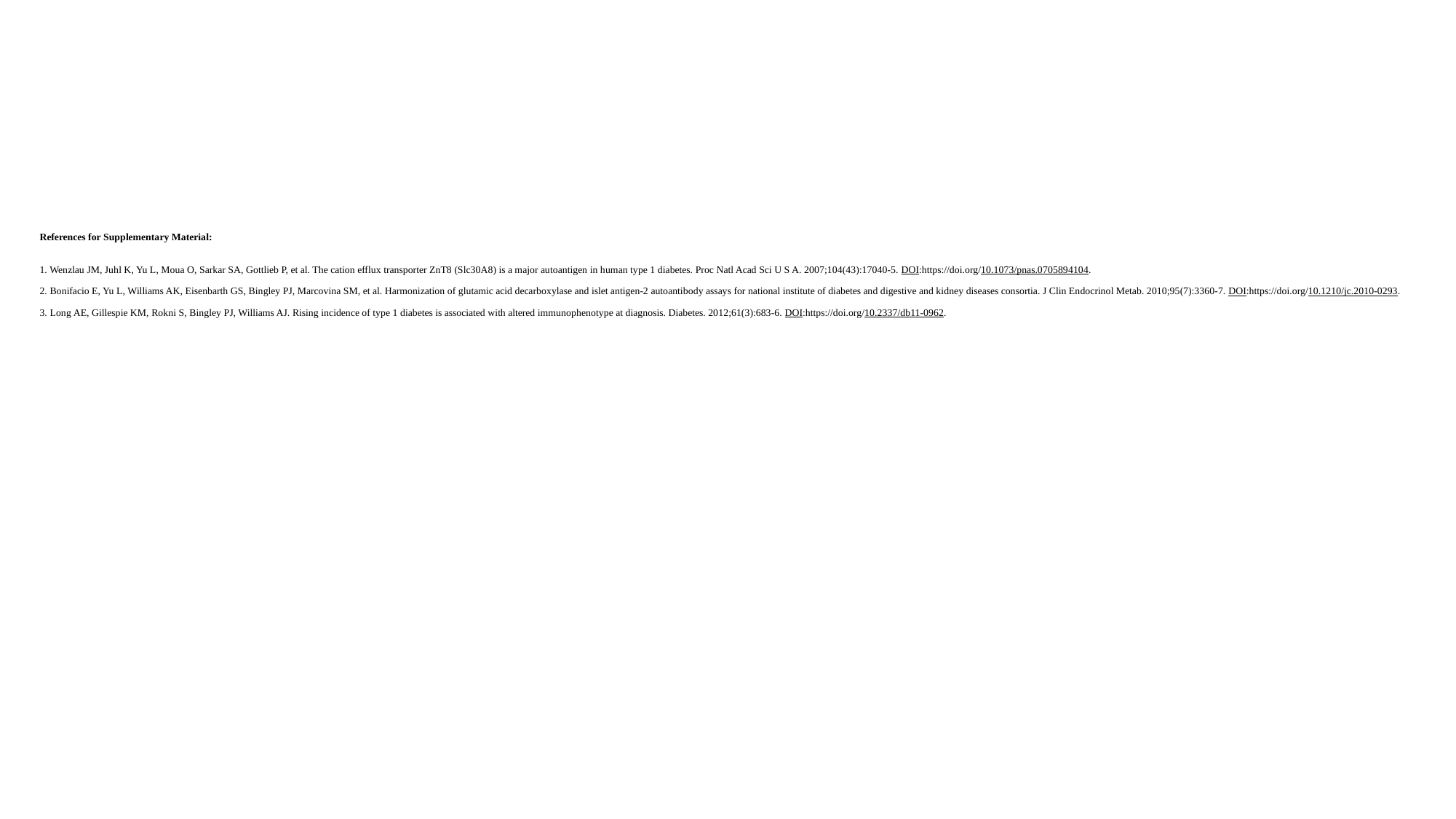

# References for Supplementary Material: 1. Wenzlau JM, Juhl K, Yu L, Moua O, Sarkar SA, Gottlieb P, et al. The cation efflux transporter ZnT8 (Slc30A8) is a major autoantigen in human type 1 diabetes. Proc Natl Acad Sci U S A. 2007;104(43):17040-5. DOI:https://doi.org/10.1073/pnas.0705894104. 2. Bonifacio E, Yu L, Williams AK, Eisenbarth GS, Bingley PJ, Marcovina SM, et al. Harmonization of glutamic acid decarboxylase and islet antigen-2 autoantibody assays for national institute of diabetes and digestive and kidney diseases consortia. J Clin Endocrinol Metab. 2010;95(7):3360-7. DOI:https://doi.org/10.1210/jc.2010-0293. 3. Long AE, Gillespie KM, Rokni S, Bingley PJ, Williams AJ. Rising incidence of type 1 diabetes is associated with altered immunophenotype at diagnosis. Diabetes. 2012;61(3):683-6. DOI:https://doi.org/10.2337/db11-0962.
